# Investigation of carbon dioxide solubility in various families of deep eutectic solvents by the PC-SAFT EoS

**DOI:** 10.3389/fchem.2022.909485

**Published:** 2022-08-09

**Authors:** Khalil Parvaneh, Reza Haghbakhsh, Ana Rita C. Duarte, Sona Raeissi

**Affiliations:** ^1^ Department of Chemical Engineering, University of Gonabad, Gonabad, Iran; ^2^ Department of Chemical Engineering, Faculty of Engineering, University of Isfahan, Isfahan, Iran; ^3^ LAQV, REQUIMTE, Departamento de Química da Faculdade de Ciências e Tecnologia, Universidade Nova de Lisboa, Caparica, Portugal; ^4^ School of Chemical and Petroleum Engineering, Shiraz University, Shiraz, Iran

**Keywords:** association schemes, DES (deep eutectic solvents), CO_2_, vapor-liquid equilibria (VLE), phase behavior

## Abstract

Having been introduced in 2003, Deep Eutectic Solvents (DESs) make up a most recent category of green solvents. Due to their unique characteristics, and also their tunable physical properties, DESs have shown high potentials for use in various applications. One of the investigated applications is CO_2_ absorption. The thermodynamic modeling of CO_2_ solubility in DESs has been pursued by a number of researchers to estimate the capacity and capability of DESs for such tasks. Among the advanced equations of state (EoSs), the Perturbed Chain-Statistical Associating Fluid Theory (PC-SAFT) is a well-known EoS. In this study, the performance of the PC-SAFT EoS for estimating CO_2_ solubility in various DESs, within wide ranges of temperatures and pressures, was investigated. A large data bank, including 2542 CO_2_ solubility data in 109 various-natured DESs was developed and used for this study. This is currently the most comprehensive study in the open literature on CO_2_ solubility in DESs using an EoS. For modeling, the DES was considered as a pseudo-component with a 2B association scheme. CO_2_ was considered as both an inert and a 2B-component and the results of each association scheme were compared. Considering the very challenging task of modeling a complex hydrogen bonding mixture with gases, the results of AARD% being lower than 10% for both of the investigated association schemes of CO_2_, showed that PC-SAFT is a suitable model for estimating CO_2_ solubilities in various DESs. Also, by proposing generalized correlations to predict the PC-SAFT parameters, covering different families of DESs, the developed model provides a global technique to estimate CO_2_ solubilities in new and upcoming DESs, avoiding the necessity of further experimental work. This can be most valuable for screening and feasibility studies to select potential DESs from the innumerable options available.

## Introduction

Global warming is one of the most important issues of this century. Since 1980, an increase of about 0.6 C in the mean temperature of the globe (both the northern and southern hemispheres) has been reported (Florides and Christodoulides, 2009). The emissions of greenhouse gases, such as carbon dioxide, methane, and nitrous oxide into the atmosphere have their impact on this environmental crisis. Among the greenhouse gases, CO_2_ plays a major role (Yamasaki, 2003; Florides and Christodoulides, 2009; Ali et al., 2014). Over the past decades, the concentration of carbon dioxide in the atmosphere has increased, partly because of industrial activities. Particularly, the burning of fossil fuels such as natural gas, petroleum, and coal in various industries causes CO_2_ emissions (Yamasaki, 2003; Li et al., 2019). Therefore, the absorption of this gas is a serious concern (Mulia et al., 2017). One of the commonly used methods is absorption by conventional solvents. However, most conventional solvents are not sustainable and have, themselves, caused environmental damage in the recent decades. Finding sustainable and environmentally-friendly solvents, which have the desired properties for CO_2_ absorption, is vital. In this respect, Deep Eutectic Solvents (DESs) are recently proposed green solvents which have also been investigated by researchers for CO_2_ absorption (Abbott et al., 2003; Koel, 2005; Wells and Coombe, 2006; Hasib-ur-Rahman et al., 2010; Vega et al., 2010; Haghbakhsh et al., 2019).

A DES is a mixture, consisting of at least two components that have the ability to establish new hydrogen bonds between the constituents. They are usually created by mixing a hydrogen bond acceptor (HBA), commonly a quaternary ammonium or phosphonium salt, and a hydrogen bond donor (HBD), such as metal salts or organics acids. DESs possess a number of desirable properties, such as having low vapor pressure, as well as being task-specific, easy to synthesize, cheap, non-flammable, sustainable, and biodegradable (Smith et al., 2014; Altamash et al., 2017; Ma et al., 2017; Haghbakhsh et al., 2019; Haider et al., 2020; Khajeh et al., 2020).

These interesting properties have led to significant investigations on DESs in various fields. Researchers have studied DES applications covering, for example, nanotechnology (Smith et al., 2014), gas absorption (Mirza et al., 2017; Marcus, 2018; Li et al., 2019), catalytic reactions (Ilgen et al., 2009), purification of biodiesels (Abbott et al., 2007), biomass treatment (Xia et al., 2014) electrochemistry (Abbott et al., 2011), and drug solubilization (Morrison et al., 2009).

Regarding the field of gas absorption, up to now, researchers have focused on the absorption of CO_2_ more than on other gases. Various DESs, having different chemical natures, have been considered for CO_2_ absorption, and wide ranges of pressures and temperatures have been studied (Li et al., 2008; Zhang et al., 2012; Leron and Li, 2013a; Leron and Li, 2013b; Francisco et al., 2013; Leron et al., 2013; Chen et al., 2014; Li et al., 2014; Zubeir et al., 2014; Lu et al., 2015; Mirza et al., 2015; Deng et al., 2016; Ji et al., 2016; Zubeir et al., 2016; Altamash et al., 2017; Ghaedi et al., 2017; Liu et al., 2017; Sarmad et al., 2017; Altamash et al., 2018; Haider et al., 2018; Li et al., 2018; Zubeir et al., 2018; Liu et al., 2019; Wang et al., 2019). These studies have accumulated a data bank for CO_2_ absorption by DESs, providing vital information for pilot or industrial planning. Although many DESs have been investigated, their number is insignificant compared to the number of DESs remaining uninvestigated. There are countless DESs and they differ greatly in nature and variation. But the experimentation process is time-consuming and enatils high expenses, and so, investigating CO_2_ solubility for all is not possible. Therefore, developing global thermodynamic models for the estimation of CO_2_ solubility in various DESs is a recommended procedure to overcome the mentioned limitations.

Besides, since DESs have been introduced only recently, few thermodynamic models have been investigated for their phase behavior with CO_2_ (Florides and Christodoulides, 2009; Zubeir et al., 2014; Mirza et al., 2015; Zubeir et al., 2016; Animasahun et al., 2017; Dietz et al., 2017; Haghbakhsh and Raeissi, 2017; Lloret et al., 2017). Simple cubic equations of state (EoSs), such as the Peng−Robinson (PR) and modified Peng−Robinson have been considered by some researchers (Florides and Christodoulides, 2009; Zubeir et al., 2014; Mirza et al., 2015). In these studies, a DES was considered as a pseudo-pure compound whose critical properties were calculated using a group-contribution procedure, namely, the modified Lyderson-Joback-Reid model (Joback and Reid, 1987). To achieve more reliable results, some researchers considered more complex models. Haghbakhsh and Raeissi (2017) used the cubic plus association (CPA) equation of state to model the solubility of CO_2_ in various DESs. They also investigated different association schemes of CO_2_ and found that the inert scheme, with fewer fitting parameters, is the most accurate. Lloret et al. (2017) applied the Soft-SAFT (Soft-Statistical Associating Fluid Theory) EoS, to describe the density, surface tension, viscosity, and phase behavior of CO_2_ with several tetraalkylammonium chloride-based DESs. They explored the two approaches of either describing the DESs as pseudo-compounds or treating them as combinations of their two independent constituents of HBA and HBD. Further, the effort was made to model CO_2_ solubility in DES systems using the PC-SAFT (Perturbed Chain-Statistical Associating Fluid Theory) EoS, as a well-known version of the SAFT type family (Zubeir et al., 2016; Dietz et al., 2017; Animasahun et al., 2017; Aminian, 2021; Cea-Klapp et al., 2020). For the first time, Zubeir et al. (2016) used the PC-SAFT to peruse the phase behavior of a few DES + CO_2_ mixtures at a temperature range between 298.15 and 318.15 K and pressures up to 2 MPa. They presented two strategies for calculating CO_2_ + DES phase behavior, where the DES was either considered as a pseudo-pure component, in which the pure parameters were calculated based on density data, or it was treated as two individual components (HBA and HBD). Dietz et al. (2017) applied the pseudo-pure approach for PC-SAFT modeling of CO_2_ phase behavior for a few hydrophobic DESs, and reported reliable results with respect to the experimental data. Animasahun et al. (2017) also used the PC-SAFT, as well as two different cubic EoSs (Peng−Robinson and Soave-Redlich-Kwong) to estimate CO_2_ solubilities in some DESs. They investigated 20 different CO_2_ + DES systems, in which choline chloride was considered as the HBA in 16 of the systems. Only the association scheme of 2B was considered for carbon dioxide. Further, Aminian (Aminian, 2021) studied the phase behavior of systems containing ionic liquids (ILs) and DESs using the PC-SAFT EoS. Their investigated DESs were based on tetrabutylammonium chloride and tetrabutylammonium bromide (as the HBA), and levulinic acid and diethylene glycol (as the HBD) at two different molar ratios. Their phase equilibrium results by PC-SAFT were compared to the COSMO-RS model. Cea-Klapp et al. (2020) also applied the PC-SAFT EoS for calculating the phase equilibria of DES systems. By using temperature-dependent binary interaction parameters, they succeeded to present more reliable results, especially in the case of liquid-liquid equilibria in ternary systems.

Despite the advancements in DES modeling, there is still the need for further studies to improve the phase behavior modelling of such complex hydrogen-bonding components with CO_2_. In this study, the largest and most comprehensive data bank of CO_2_ solubility in various families of DESs was developed, which is much more extended and generalized than any previous study. The experimental densities of DESs are used to optimize the pure PC-SAFT parameters of the DESs with different chemical natures. The phase behavior of carbon dioxide with various DESs is then modelled with the PC-SAFT EoS over wide ranges of pressures and temperatures. Two feasible association schemes of inert and 2B are considered for carbon dioxide, which has not yet been investigated for the PC-SAFT EoS. Furthermore, to make the model predictive, and so, suitable for feasibility and screening studies on DESs for carbon capture applications, generalized correlations are proposed for estimation of the PC-SAFT parameters.

## Theoretical background

### The perturbed-chain SAFT (PC-SAFT) equation of state

Gross and Sadowski (Gross and Sadowski, 2001; Gross and Sadowski, 2002) proposed the PC-SAFT equation of state based on the combination of different terms of the reduced Helmholtz energy. These terms are the reduced residual Helmholtz energy (*a͂* ^*res*^), the reduced hard-chain Helmholtz energy (*a͂* ^*hc*^), the reduced dispersion Helmholtz energy (*a͂* ^*disp*^), and the reduced associating contribution Helmholtz energy (*a͂* ^*assoc*^), which is presented as follows.

$${\tilde{a}}^{res}={\tilde{a}}^{hc}+{\tilde{a}}^{disp}+{\tilde{a}}^{assoc}$$ (1)

The reduced hard-chain Helmholtz energy is expressed through (Eqs 2–(4) (Gross and Sadowski, 2001; Gross and Sadowski, 2002; Parvaneh and Shariati, 2017; Haghbakhsh et al., 2018a; Haghbakhsh et al., 2018b).

$${\tilde{a}}^{hc}=\bar{m}{\tilde{a}}^{hs}-\sum_{i}x_{i}\left(m_{i}-1\right)\ln g_{ii}^{hs}$$ (2)

$${\tilde{a}}^{hs}=\frac{1}{\zeta_{0}}\left[\frac{3\zeta_{1}\zeta_{2}}{1-\zeta_{3}}+\frac{\zeta_{2}^{3}}{\zeta_{3}{\left(1-\zeta_{3}\right)}^{2}}+\left(\frac{\zeta_{2}^{3}}{\zeta_{3}^{2}}-\zeta_{0}\right)ln\left(1-\zeta_{3}\right)\right]$$ (3)

$$g_{ij}^{hs}=\frac{1}{1-\zeta_{3}}+\frac{d_{i}d_{j}}{d_{i}+d_{j}}\frac{3\zeta_{2}}{{\left(1-\zeta_{3}\right)}^{2}}+{\left(\frac{d_{i}d_{j}}{d_{i}+d_{j}}\right)}^{2}\frac{2\zeta_{2}^{2}}{{\left(1-\zeta_{3}\right)}^{3}}$$ (4)

where *a͂* ^*hs*^, *m*_*i*_, *x*_*i*_, and *g*_*ij*_^*hs*^ are the reduced Helmholtz energy of the hard sphere, the number of segments, chain mole fraction, and radial pair distribution function, respectively. *d*_*i*_ is the temperature-dependent hard segment diameter for component *i,* which is calculated using the following equations (Gross and Sadowski, 2001; Gross and Sadowski, 2002; Parvaneh and Shariati, 2017; Haghbakhsh et al., 2018a; Haghbakhsh et al., 2018b).

$$d_{i}=\sigma_{i}\left[1-0.12exp\left(-\frac{3\varepsilon_{i}}{k_{B}T}\right)\right]$$ (5)

$$\zeta_{n}=\frac{\pi }{6}\rho \sum_{i}x_{i}m_{i}d_{i}^{n}\;n\in \left\{0,1,2,3\right\}$$ (6)

where *m*_*i*_ (number of the segment), *σ*_*i*_ (segment diameter), and *ε*_*i*_ */k*_*B*_ (segment energy) are the pure component parameters that should be optimized based on experimental data.

The functionality of the dispersion (*a͂* ^*disp*^) and associating (*a͂* ^*assoc*^) contributions are given through the following equations (Gross and Sadowski, 2001; Gross and Sadowski, 2002; Parvaneh and Shariati, 2017; Haghbakhsh et al., 2018a; Haghbakhsh et al., 2018b).

$${\tilde{a}}^{disp}=-2\pi \rho I_{1}\bar{m^{2}\varepsilon \sigma^{3}}-\pi \rho \bar{m}C_{1}I_{2}\bar{m^{2}\varepsilon^{2}\sigma^{3}}$$ (7)

$${\tilde{a}}^{assoc}=\sum_{i}x_{i}\sum_{A_{i}}\left(lnX^{A_{i}}-\frac{X^{A_{i}}}{2}+\frac{M_{i}}{2}\right)$$ (8)

$$X^{A_{i}}={\left(1+\rho \sum_{j}x_{j}\sum_{Bj}X^{B_{j}}\Delta^{A_{i}B_{j}}\right)}^{-1}$$ (9)

$$\Delta^{A_{i}B_{j}}=g_{ij}^{seg}\kappa^{A_{i}B_{j}}\sigma_{ij}^{3}\left(exp\left(\frac{\varepsilon^{A_{i}B_{j}}}{kT}\right)-1\right)$$ (10)

where *X* ^*Ai*^, *M*_*i,*_ and *Δ* ^*AiBj*^ are the mole fraction of component *i* that is not bonded at site *A*, the number of association sites, and the association strength, respectively. In addition to *m*_*i*_, *σ*_*i,*_ and *ε*_*i*_ */k*_*B*_, the effective association volume (*κ* ^*AiBi*^) and association energy (*ε* ^*AiBi*^ */k*) are also pure compound constants that must be considered for associating compounds.

### Investigated compounds

In this research, the largest data bank, up to date, of CO_2_ solubility in DESs, consisting of 109 various natured DESs, was collected from the open literature. This data bank consists of 2,542 data points, covering wide ranges of pressures and temperatures. Supplementary Table S1 (Supplementary Material) presents the pressure, temperature, and CO_2_ solubility ranges of the investigated DESs in this study. The corresponding literature reference of each DESs is also given in Supplementary Table S1 (Li et al., 2008; Leron and Li, 2013a; Leron and Li, 2013b; Francisco et al., 2013; Leron et al., 2013; Chen et al., 2014; Li et al., 2014; Zubeir et al., 2014; Lu et al., 2015; Mirza et al., 2015; Deng et al., 2016; Ji et al., 2016; Zubeir et al., 2016; Altamash et al., 2017; Ghaedi et al., 2017; Liu et al., 2017; Sarmad et al., 2017; Altamash et al., 2018; Haider et al., 2018; Li et al., 2018; Zubeir et al., 2018; Liu et al., 2019; Wang et al., 2019). In the case of those limited DESs for which solubility data were presented by more than one research group, no discriminations were carried out and all of the data by all groups were considered in the development of the model.

## Results and discussion

The pseudo-component approach was used for modeling CO_2_ + DES systems, which is a well-known model having been used for various DESs (Zubeir et al., 2016; Dietz et al., 2017; Lloret et al., 2017). The association scheme of 2B, as presented by Huang and Radosz (Huang and Radosz, 1990; Huang and Radosz, 1991), was considered for the pseudo-component DESs. The reason for considering the pseudo-component approach, and also choosing the 2B association scheme for DESs is by following the recommendations of many published studies (Zubeir et al., 2016; Haghbakhsh and Raeissi, 2017; Lloret et al., 2017; Dietz et al., 2017; Animasahun et al., 2017; Aminian, 2021; Cea-Klapp et al., 2020). Almost all of the literature that have used the PC-SAFT EoS for modeling of DESs, have recommended the 2B association scheme for a pseudo-component DES. The schematic representation of the considered association schemes of the investigated systems are presented as Figure S1 of the Supplementary Material. For CO_2_, the inert and 2B association schemes are the most commonly used association schemes in the literature, as indicated by the studies of Haghbakhsh and Raeissi, and also Baramaki et al. (Haghbakhsh and Raeissi, 2017; Baramaki et al., 2019). Therefore, we chose to investigate and compare both of these schemes in this study.

To model the phase behavior of CO_2_ + DES systems, the first calculation step was to estimate the PC-SAFT pure parameters of *m*_*i*_ (segment number), *σ*_*i*_ (segment diameter), *u*_*i*_ */k* (segment energy parameter), *κ* ^*AiBj*^ (effective association volume), and *ε* ^*AiBj*^ */k* (association energy). These five parameters are optimized to the liquid density data of the DESs based on Eq. (11) as the objective function.

$$OF={\sum_{i}^{Np}\left(\frac{\rho_{i}^{\exp.}-\rho_{i}^{calc.}}{\rho_{i}^{\exp.}}\right)}^{2}$$ (11)

in which, *ρ*_*i*_^*exp.*^ and *ρ*_*i*_^*calc.*^ are the experimental and calculated density, respectively, and *Np* is the number of the data points.

However, in order to reduce the number of adjustable parameters of the PC-SAFT EoS for DESs, the effective association volume (*κ* ^*AiBj*^) and association energy (*ε* ^*AiBj*^ */k*) parameters were considered as 0.1 and 5,000, respectively (Zubeir et al., 2016; Dietz et al., 2017).

The number of literature density data for each DES, the range of liquid densities, and the corresponding reference are given in Supplementary Table S2. However, for some of the investigated DESs, no density data have been reported in the open literature. Thus, for those DESs, distinguished in Supplementary Table S3, the general density model of Haghbakhsh et al. (Haghbakhsh et al., 2019) was used to generate the density data. Haghbakhsh et al.’s density model (Haghbakhsh et al., 2019) is a function of temperature, critical volume, critical temperature, and acentric factor of the DES pseudo-component. The modified Lydersen-Joback-Reid approach (Valderrama and Robles, 2007; Valderrama et al., 2008) and the Lee-Kesler mixing rules, which were presented by Knapp et al. (1982), were used to calculate the critical properties and acentric factors of the DESs that lacked density data. The results are presented in Supplementary Table S4 of the Supplementary material.

The three pure component parameters of PC-SAFT, optimized to the collected density data of the investigated DESs, are presented in Table 1. For some of the investigated DESs, the values of the PC-SAFT parameters were previously given in published studies, and in these cases, the literature values were considered, as reported in Table 1. For CO_2_, the values of the three pure component PC-SAFT parameters for both the association schemes of 2B and inert have been reported in the literature, and these values are also presented in Table 1. Furthermore, the effective association volume (*κ* ^*AiBj*^) and association energy (*ε* ^*AiBj*^ */k*) parameters of CO_2_ for the association schemes of 2B are 0.03318 and 576.7, respectively (Baramaki et al., 2019).

**TABLE 1:** The values of optimized PC-SAFT parameters for the investigated DESs in this study and carbon dioxide^b^.

Abbr.	*m*_*i*_	*σ*_*i*_ (*Ǻ*)	*ɛ*_*i*_ */k* (*K*)	*AARD %*
DES1	3.6803	3.6018	420.74	0.26
DES2	9.4497	2.6755	309.65	0.28
DES3	8.9837	2.7142	327.52	0.23
DES4	7.0125	2.9335	340.12	0.23
DES5	10.3775	2.4586	343.99	0.44
DES6	9.3189	2.4892	336.29	0.4
DES7	8.7806	2.4332	339.50	0.53
DES8	9.7048	2.6265	318.15	0.09
DES9	3.2933	3.1822	381.91	0.15
DES10	3.4444	3.2491	355.21	0.17
DES11	3.4128	3.8870	335.16	0.05
DES12	3.4170	3.6864	358.76	0.02
DES13	3.3869	3.6223	354.27	0.03
DES14	3.5068	3.8034	332.58	0.06
DES15	3.4600	3.6919	332.86	0.13
DES16	3.5507	4.1042	327.82	0.05
DES17	3.5021	3.9730	327.72	0.02
DES18	3.4751	3.9429	332.31	0.03
DES19	7.5169	2.7308	359.28	0.69^a^
DES20	6.9359	2.8935	349.74	0.67^a^
DES21	7.1671	2.8281	342.91	0.59^a^
DES22	6.6122	2.8056	380.24	0.57^a^
DES23	5.7468	3.04907	407.43	0.63^a^
DES24	3.1064	3.4753	387.70	0.15
DES25	3.4218	3.4959	395.86	0.12
DES26	3.3837	3.3711	348.92	0.05
DES27	4.3270	3.0785	350.00	0.11
DES28	4.3312	3.2648	391.45	0.17
DES29	3.6904	3.4178	390.63	0.17
DES30	4.6418	3.2047	393.62	0.10
DES31	5.3061	3.0173	332.52	0.10
DES32	6.6024	3.1179	348.71	0.70^a^
DES33	7.8434	2.6487	356.71	0.66^a^
DES34	6.3107	2.9103	345.30	0.30
DES35	4.6314	3.2144	361.14	0.22
DES36	3.7883	3.1277	320.39	0.30
DES37	6.2709	3.1457	368.99	0.69^a^
DES38	8.4619	2.5881	345.22	0.14
DES39	7.5213	2.6746	347.78	0.08
DES40	6.7674	2.7601	354.09	0.07
DES41	5.3496	2.9241	310.00	0.42
DES42	9.1060	2.6631	351.98	0.21
DES43	7.0991	2.8825	352.35	0.08
DES44	5.5588	3.1125	341.30	0.08
DES45	6.1204	2.9585	396.20	0.60^a^
DES46	5.1851	2.9327	284.13	0.62
DES47	5.4774	3.0834	323.72	0.10
DES48	3.7486	3.4475	291.86	0.08
DES49	3.0017	3.6851	280.52	0.09
DES50	6.3275	3.0243	377.26	0.66^a^
DES51	7.1170	2.8502	347.81	0.62^a^
DES52	7.1606	2.845	355.33	0.62^a^
DES53	6.3374	2.8215	257.25	0.24
DES54	5.3589	2.9327	241.00	0.26
DES55	5.1909	2.9327	229.25	0.24
DES56	9.0710	2.8000	330.58	0.15
DES57	8.7878	2.8324	317.32	0.24
DES58	3.4365	3.2789	353.06	0.49^a^
DES59	3.3586	3.1858	360.18	0.27
DES60	3.3674	3.1902	383.90	0.68^a^
DES61	10.5724	2.5122	323.10	0.15
DES62	10.5806	2.5346	362.07	0.17
DES63	10.5485	2.5390	361.91	0.19
DES64	11.0243	3.3621	304.89	0.07
DES65	11.0359	3.3701	305.57	0.07
DES66	6.3126	3.0152	372.90	0.64^a^
DES67	6.0238	2.9766	358.31	0.61^a^
DES68	6.8842	2.9122	387.74	0.24
DES69	9.5777	2.6250	347.43	0.26
DES70	6.7541	3.2009	342.42	0.67^a^
DES71	6.3977	3.1206	378.56	0.54^a^
DES72	7.8481	3.5327	337.11	0.06
DES73	11.7300	2.6760	303.76	Zubeir et al. (2016)
DES74	10.5328	2.6760	338.00	Zubeir et al. (2016)
DES75	8.7261	2.9327	266.40	0.08
DES76	8.9204	2.8600	330.34	0.61^a^
DES77	7.2637	3.0673	366.66	0.60^a^
DES78	6.8383	2.9416	341.56	0.60^a^
DES79	6.4777	2.9094	387.90	0.60^a^
DES80	6.1411	3.0882	357.80	0.61^a^
DES81	9.5816	2.9376	321.28	0.65^a^
DES82	8.3764	2.9603	339.72	0.64^a^
DES83	7.2833	3.0302	357.95	0.63^a^
DES84	6.4463	2.7725	356.45	0.58^a^
DES85	6.4289	2.7432	382.36	0.58^a^
DES86	8.6644	2.9327	252.88	0.14
DES87	3.9823	3.7122	365.60	0.02
DES88	4.0299	3.7590	364.79	0.06
DES89	8.0400	2.5831	329.75	0.57^a^
DES90	7.2645	2.5935	365.81	0.56^a^
DES91	3.2607	3.7539	599.88	Zubeir et al. (2016)
DES92	5.5147	3.1379	280.15	0.10
DES93	8.8536	2.8989	344.45	0.60^a^
DES94	5.2004	3.2659	350.74	0.05
DES95	4.9495	2.7400	379.67	0.48^a^
DES96	2.9824	3.5678	506.01	Zubeir et al. (2016)
DES97	15.4820	3.1583	317.42	Dietz et al. (2017)
DES98	14.8000	3.2400	382.09	Dietz et al. (2017)
DES99	15.3220	3.1533	307.11	Dietz et al. (2017)
DES100	7.2315	2.5414	359.78	0.58^a^
DES101	6.1747	2.7751	352.67	0.55^b^
DES102	5.8932	2.6729	340.80	0.56^a^
DES103	6.5848	2.7365	369.45	0.53^a^
DES104	6.2763	2.7635	364.94	0.55^a^
DES105	6.8388	2.7936	353.41	0.63^a^
DES106	6.2883	2.9043	396.64	0.60^a^
DES107	6.7412	2.9691	359.07	0.60^a^
DES108	3.7112	3.3667	404.08	0.29
DES109	8.9137	2.6324	335.54	0.10
CO_2_ (inert)	2.0729	2.7852	169.21	Gross and Sadowski, (2001)
CO_2_ (2B)^c^	2.1051	2.7841	162.08	Baramaki et al. (2019)

a Density data was obtained according to Haghbakhsh et al.‘s density model (Haghbakhsh et al., 2019).

b The values of the (*κ* ^*AiBj*^) and (*ε* ^*AiBj*^ */k*) parameters were considered as 0.1 and 5,000, respectively, for all of the investigated DESs (Zubeir et al., 2016; Dietz et al., 2017).

c The values of the (*κ* ^*AiBj*^) and (*ε* ^*AiBj*^ */k*) parameters of CO_2_ for the association schemes of 2B are 0.03318 and 576.7, respectively (Baramaki et al., 2019).

In order to check the consistency of the values of the calculated critical properties and the optimized PC-SAFT parameters for the investigated DESs, the thermodynamic evaluation of the global map of Polishuk et al. (2013) has been carried out. For this purpose, values for the reduced critical temperatures have been calculated according to Eq. (12). Since these values were calculated to be greater than 1 for all of the DESs, according to the thermodynamic analysis of Polishuk et al. (2013), no unrealistic values have been found for the calculated critical properties and optimized PC-SAFT parameters. The details of this evaluation are given in Supplementary Table S5 of the Supplementary Material.

Tc∗=Tc(εk) (12)

**FIGURE 1:**
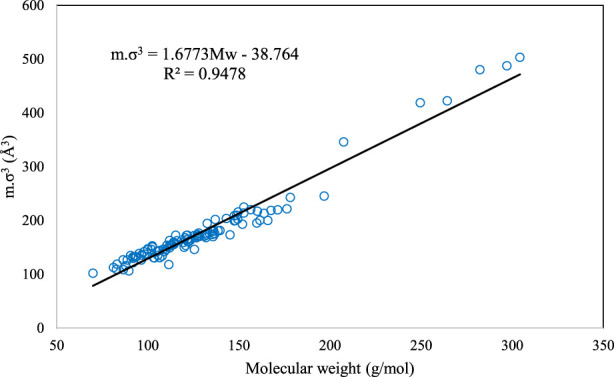
The behavior of *m.σ* ^*3*^ for PC-SAFT with molecular weight (Mw) for all of the investigated DESs in this study.

Also, in order to have a more comprehensive investigation and provide predictive ability for the PC-SAFT model, a generalized correlation for the estimation of the PC-SAFT pure component parameters of the studied DESs was developed in this work, as presented by Eq. (13).

$$m\sigma^{3}=1.6773MW-38.764$$ (13)

where is *m* is the segment number and *σ* is the segment diameter, which are related to the molecular weight, *MW*, of the DES. The graphical behavior of the developed generalized correlation for the studied DESs is depicted in Figure 1. In this figure, the relations of *mσ* ^*3*^ of the PC-SAFT EoS with respect to the molecular weight are shown for all 109 investigated DESs. As can be seen, *mσ* ^*3*^ has an increasing behavior with increasing molecular weight. Despite the simplicity, this correlation succeeds to consider various DESs, having very different natures, with high accuracy.

Although the above correlation is global and can be used for all the DESs, we have also provided two family-specific correlations for higher accuracy. For the families of 1-choline chloride + n-levulinic acid (n = 3, 4, 5) and 1-tetrabutyl ammonium bromide + n-diethylene glycol (n = 2, 3, 4) specific correlations were developed and presented as Eqs (14), (15), respectively.

$$m\sigma^{3}=5.3361MW-490.57$$ (14)

$$m\sigma^{3}=1.3983MW-6.3921$$ (15)

**FIGURE 2:**
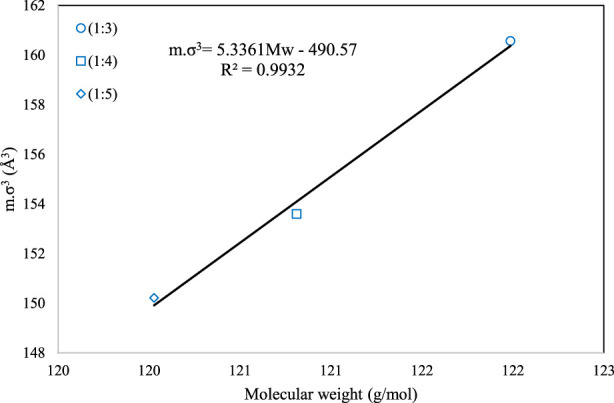
The behavior of *m.σ* ^*3*^ for PC-SAFT with molecular weight (Mw) for the 1-choline chloride + n-levulinic acid family [n = 3(o), 4 (**□**) and 5 (**⋄**)].

**FIGURE 3:**
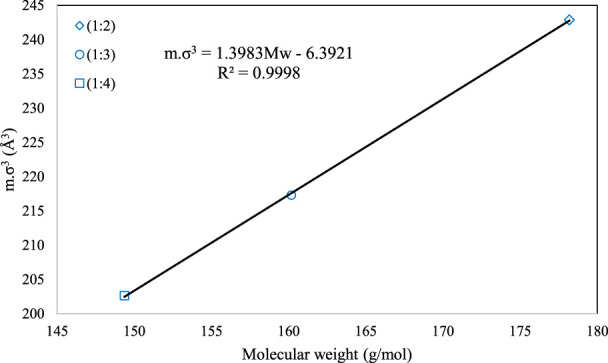
The behavior of *m.σ* ^*3*^ for PC-SAFT with molecular weight (Mw) for the 1-tetrabutyl ammonium bromide + n-diethylene glycol family [n = 2(o), 3 (**□**) and 4 (**⋄**)].

Figures 2, 3 present the graphical behavior of these two specific family correlations with respect to molecular weight. As one would expect, one single generalized equation, correlated with only the molecular weight for all types of DESs with different natures and different molar ratios, is not as accurate (Figure 1) as the family-specific correlations, with constants that are fine-tuned to the particular structural family (Figures 2, 3).

Also, in order to investigate the predictive ability of the PC-SAFT model, CO_2_ solubilities were calculated according to the two modes of prediction and correlation of PC-SAFT. In the prediction mode, the solubilities were achieved without considering any binary interaction parameters (*k*_*ij*_), while in the correlation mode, binary interaction parameters were considered according to the following equation, with the purpose to correct the segment-segment interactions of dissimilar chains (Gross and Sadowski, 2001).

$$\varepsilon_{ij}=\sqrt{\varepsilon_{i}\varepsilon_{j}}\left(1-k_{ij}\right)$$ (16)

To involve the effect of temperature on the binary interaction parameters, the temperature functionality of Eq. (17) was taken into account.

$$k_{ij}=a+b\times T$$ (17)

The two adjustable parameters (*a* and *b*) were optimized based on the CO_2_ solubility data in various DESs using the following objective function, and the values are reported in Table 2.

$$OF={\sum_{i}^{N_{P}}\left(\frac{x_{i}^{Exp.}-x_{i}^{Calc.}}{x_{i}^{Exp.}}\right)}^{2}$$ (18)

**TABLE 2:** The values of optimized parameters and *AARD%* for the two predictive and correlative modes of PC-SAFT EoS by considering two association schemes for CO_2_.

	Inert (CO_2_)+ 2B (DES)				2B (CO_2_) + 2B (DES)			
	Predictive	Correlative			Predictive	Correlative		
	k_ij_ = 0	k_ij=_a+b.T			k_ij_ = 0	k_ij=_a+b.T		
	AARD%	AARD%	a	b	AARD%	AARD%	a	b
DES1	91.22	44.48	0.00013	1.53 × 10^−4^	126.56	44.48	0.00015	2.19 × 10^−4^
DES2	64.98	1.61	−0.11915	2.22 × 10^−4^	55.74	1.57	−0.11216	2.35 × 10^−4^
DES3	74.24	1.87	-0.11839	1.75 × 10^−4^	67.35	1.85	-0.11027	1.84 × 10^−4^
DES4	47.11	1.62	−0.07758	1.43 × 10^−4^	30.64	1.57	−0.06605	1.51 × 10^−4^
DES5	96.54	7.35	−0.27373	4.27 × 10^−4^	95.75	7.37	−0.27141	4.43 × 10^−4^
DES6	94.78	4.50	-0.26806	4.49 × 10^−4^	93.38	4.56	-0.26533	4.70 × 10^−4^
DES7	97.22	8.75	−0.30329	4.97 × 10^−4^	96.40	8.85	−0.30089	5.19 × 10^−4^
DES8	84.25	3.03	−0.28205	6.06 × 10^−4^	80.35	3.07	−0.27692	6.22 × 10^−4^
DES9	50.18	15.13	0.18890	−7.15 × 10^−4^	23.12	15.10	0.21407	−7.15 × 10^−4^
DES10	35.17	16.54	0.22557	−7.79 × 10^−4^	27.00	17.02	0.25662	−7.91 × 10^−4^
DES11	15.97	12.60	0.01029	0	36.44	11.99	0.03583	0
DES12	14.13	9.89	−0.01059	0	18.28	9.17	0.01403	0
DES13	12.62	12.19	−0.00483	0	28.35	11.50	0.02027	0
DES14	23.36	8.22	0.00028	6.12 × 10^−5^	59.81	7.97	0.00017	1.37 × 10^−4^
DES15	10.69	4.84	0.02300	−4.94 × 10^−5^	46.84	4.78	0.04894	−4.82 × 10^−5^
DES16	29.48	10.83	0.03073	0	55.82	10.33	0.05537	0
DES17	40.11	8.17	0.03675	0	75.15	4.64	0.06205	0
DES18	58.86	11.08	0.04603	0	100.48	10.60	0.07128	0
DES19	89.24	9.00	-0.09664	0	85.68	8.62	-0.08488	0
DES20	47.49	46.35	0.01535	0	51.00	46.26	0.02943	0
DES21	75.08	5.62	−0.06410	0	66.55	5.43	−0.05068	0
DES22	92.91	21.23	−0.11819	0	90.35	20.76	−0.10513	0
DES23	90.59	17.11	−0.10412	0	87.14	16.58	−0.09010	0
DES24	27.27	13.94	0.22373	-6.74 × 10^−4^	69.48	13.93	0.25247	-6.81 × 10^−4^
DES25	14.51	10.08	0.23499	-7.41 × 10^−4^	31.66	10.15	0.27122	-7.77 × 10^−4^
DES26	242.5	6.73	0.00015	2.53 × 10^−4^	401.24	5.85	0.00017	3.41 × 10^−4^
DES27	41.47	3.69	−0.16969	6.13 × 10^−4^	105.49	3.63	−0.15445	6.33 × 10^−4^
DES28	46.44	2.35	−0.08064	3.33 × 10^−4^	105.76	2.38	−0.06356	3.41 × 10^−4^
DES29	175.43	3.72	0.00023	2.00 × 10^−4^	292.64	3.59	0.00466	2.57 × 10^−4^
DES30	10.77	2.37	0.01453	-3.14 × 10^−5^	54.45	2.35	0.03053	-2.42 × 10^−5^
DES31	7.95	2.54	−0.00028	1.50 × 10^−5^	50.52	2.51	0.00026	7.44 × 10^−5^
DES32	65.88	11.30	0.13579	-6.05 × 10^−4^	55.84	11.70	0.16771	-6.54 × 10^−4^
DES33	96.49	12.59	−0.14354	0	95.36	12.43	−0.13270	0
DES34	42.47	4.26	−0.16328	4.35 × 10^−4^	22.15	4.19	−0.15409	4.55 × 10^−4^
DES35	51.48	3.98	−0.10102	4.04 × 10^−4^	111.83	3.89	−0.08494	4.16 × 10^−4^
DES36	69.60	14.24	−0.20154	7.22 × 10^−4^	150.62	13.36	−0.17527	7.21 × 10^−4^
DES37	70.39	12.11	0.00014	-1.99 × 10^−4^	60.92	12.61	0.01277	-1.88 × 10^−4^
DES38	90.09	1.64	−0.19128	2.78 × 10^−4^	87.24	1.64	−0.18509	2.92 × 10^−4^
DES39	86.14	1.87	−0.17459	2.60 × 10^−4^	81.77	1.84	−0.16806	2.77 × 10^−4^
DES40	81.74	1.32	−0.13052	1.52 × 10^−4^	75.6	1.28	−0.12166	1.67 × 10^−4^
DES41	38.08	4.69	−0.25403	6.93 × 10^−4^	16.00	5.85	−0.21010	6.25 × 10^−4^
DES42	87.82	1.63	−0.20774	3.74 × 10^−4^	84.68	1.62	−0.20266	3.88 × 10^−4^
DES43	60.82	1.46	−0.12685	2.59 × 10^−4^	48.62	1.45	−0.11834	2.75 × 10^−4^
DES44	17.08	2.00	−0.06124	2.28 × 10^−4^	58.75	1.95	−0.04801	2.43 × 10^−4^
DES45	90.95	14.08	1.07 × 10–5	-3.51 × 10^−4^	87.80	14.17	0.03143	-4.06 × 10^−4^
DES46	92.22	1.95	−0.12822	5.24 × 10^−4^	173.20	2.12	−0.12631	5.90 × 10^−4^
DES47	8.23	2.17	−0.13986	4.49 × 10^−4^	41.11	2.13	−0.12790	4.70 × 10^−4^
DES48	183.48	2.90	−0.07176	4.90 × 10^−4^	291.85	2.91	−0.04673	4.98 × 10^−4^
DES49	268.21	1.56	−0.06274	5.73 × 10^−4^	408.56	1.51	−0.03684	5.94 × 10^−4^
DES50	80.55	16.73	−0.08185	1.16 × 10^−6^	74.09	17.04	−0.05477	-3.92 × 10^−5^
DES51	93.37	10.13	−0.13390	0	91.19	10.45	−0.12079	0
DES52	95.00	2.95	−0.14492	0	93.37	2.70	−0.13232	0
DES53	59.52	13.15	0.09203	-1.99 × 10^−4^	123.75	12.14	0.08996	-1.24 × 10^−4^
DES54	148.94	10.05	−0.03571	3.35 × 10^−4^	253.23	9.19	−0.01823	3.59 × 10^−4^
DES55	179.70	11.61	0.00036	2.54 × 10^−4^	298.03	10.60	0.00030	3.45 × 10^−4^
DES56	62.22	4.11	−0.18903	4.56 × 10^−4^	52.72	4.03	−0.18175	4.68 × 10^−4^
DES57	43.85	5.20	−0.15278	3.96 × 10^−4^	29.18	5.14	-0.14439	4.08 × 10^−4^
DES58	65.43	9.33	-0.06193	3.09 × 10^−4^	146.66	11.14	1.35 × 10–4	2.06 × 10^−4^
DES59	31.56	16.34	0.00023	-6.22 × 10^−5^	20.99	18.70	0.10159	-2.87 × 10^−4^
DES60	9.61	9.17	5.31 × 10–5	-3.67 × 10^−6^	45.95	10.70	3.12 × 10–4	7.80 × 10^−5^
DES61	91.19	1.83	−0.15568	1.72 × 10^−4^	89.07	1.86	−0.15005	1.81 × 10^−4^
DES62	97.44	1.42	−0.20506	1.96 × 10^−4^	96.89	1.38	−0.20029	2.02 × 10^−4^
DES63	97.47	1.05	−0.20657	1.98 × 10^−4^	96.92	1.06	−0.20309	2.08 × 10^−4^
DES64	39.39	0.98	−0.02250	1.52 × 10^−4^	59.71	0.98	−0.00839	1.38 × 10^−4^
DES65	38.21	1.47	0.00616	5.60 × 10^−5^	57.72	1.47	0.01427	6.30 × 10^−5^
DES66	44.22	30.06	−0.03073	0	34.47	29.77	−0.01633	0
DES67	77.22	28.16	−0.06353	0	68.69	27.76	−0.04826	0
DES68	73.02	9.81	−0.05605	0	64.25	9.98	−0.04362	0
DES69	78.22	39.33	−0.04963	0	72.55	39.23	−0.04048	0
DES70	125.55	45.05	0.07112	0	192.68	44.96	0.08641	0
DES71	67.71	4.10	−0.05765	0	57.26	3.72	−0.04374	0
DES72	41.75	1.00	-0.02222	1.52 × 10^−4^	68.61	1.17	−0.01099	1.57 × 10^−4^
DES73	74.86	2.56	−0.13172	2.05 × 10^−4^	69.74	2.32	−0.12671	2.17 × 10^−4^
DES74	89.89	2.33	−0.16478	1.98 × 10^−4^	87.67	2.08	−0.15906	2.07 × 10^−4^
DES75	15.98	2.77	−0.13366	4.53 × 10^−4^	44.26	2.78	−0.12424	4.71 × 10^−4^
DES76	69.09	10.47	−0.06013	0	60.87	10.34	-0.04888	0
DES77	40.12	4.27	−0.02607	0	23.16	4.17	−0.01340	0
DES78	45.95	4.68	−0.03197	0	26.96	4.61	−0.01747	0
DES79	81.79	1.70	−0.07782	0	75.60	1.58	−0.06490	0
DES80	67.29	9.38	−0.05996	0	56.25	9.26	−0.04474	0
DES81	40.80	12.19	−0.02841	0	27.18	12.11	−0.01752	0
DES82	61.42	9.00	−0.04933	0	51.33	8.89	−0.03764	0
DES83	71.14	3.85	−0.06241	0	62.79	3.78	−0.04975	0
DES84	95.41	14.63	−0.13818	0	93.64	14.61	−0.12323	0
DES85	98.21	13.43	−0.16905	0	97.53	13.69	−0.15537	0
DES86	45.79	1.75	−0.11529	4.44 × 10^−4^	82.85	1.77	−0.10450	4.61 × 10^−4^
DES87	17.84	7.31	7.42 × 10–5	4.00 × 10^−5^	52.84	7.08	0.00426	9.32 × 10^−5^
DES88	15.82	10.54	0.10802	-3.10 × 10^−4^	44.85	10.29	0.13815	−3.39 × 10^−4^
DES89	91.90	3.87	−0.10463	0	89.12	3.54	−0.09300	0
DES90	96.84	18.06	−0.13761	0	95.70	17.60	−0.12629	0
DES91	8.68	2.86	-0.04545	1.58 × 10^−4^	50.73	2.92	-0.03230	1.70 × 10^−4^
DES92	73.21	2.78	−0.09120	4.09 × 10^−4^	132.18	2.80	−0.07432	4.23 × 10^−4^
DES93	78.21	10.00	−0.07712	0	72.60	9.57	−0.06653	0
DES94	19.71	2.07	−0.10140	3.56 × 10^−4^	59.39	2.12	−0.08789	3.71 × 10^−4^
DES95	92.74	14.00	−0.11066	0	89.09	13.41	−0.09404	0
DES96	46.50	3.16	3.20 × 10–5	6.47 × 10^−5^	113.55	3.12	0.02309	5.85 × 10^−5^
DES97	27.87	0.59	−0.05763	1.20 × 10^−4^	21.06	0.62	−0.05198	1.20 × 10^−4^
DES98	72.06	1.83	−0.11720	1.51 × 10^−4^	69.84	2.03	−0.11460	1.54 × 10^−4^
DES99	17.67	0.70	−0.04276	9.70 × 10^−5^	9.53	0.58	−0.03989	1.08 × 10^−4^
DES100	98.39	4.34	−0.15918	0	97.79	4.26	−0.14756	0
DES101	89.66	7.78	−0.09991	0	85.44	7.94	−0.08463	0
DES102	97.06	20.36	−0.16026	0	95.72	21.77	−0.14326	0
DES103	92.33	10.77	−0.10894	0	89.43	10.33	−0.09581	0
DES104	90.17	13.43	−0.10066	0	86.31	13.51	−0.08664	0
DES105	55.88	23.02	−0.03057	0	40.09	22.88	−0.01694	0
DES106	83.20	5.12	−0.07731	0	77.19	5.11	−0.06412	0
DES107	64.43	3.60	−0.05155	0	52.60	3.75	−0.03774	0
DES108	21.75	5.60	0.01153	0	83.18	4.95	0.03643	0
DES109	87.83	2.98	−0.09378	0	84.13	2.20	−0.08230	0
**Total**	**63.77**	**8.08**			**84.65**	**8.12**		

The results of PC-SAFT modeling for CO_2_ solubilities in the investigated DESs were estimated and the errors were calculated by calculating *AARD%*, as given by Eq. (19).

$$AARD\%=\frac{100}{Np}\sum_{i}^{N_{P}}\left|\frac{x_{i}^{Exp.}-x_{i}^{Calc.}}{x_{i}^{Exp.}}\right|$$ (19)

in which *x*_*i*_^*Exp.*^ and *x*_*i*_^*Calc.*^ are the experimental and calculated carbon dioxide solubilities, respectively, and *N*_*P*_ is the total number of data. Table 2 presents the calculated *AARD%* values for both the correlative and predictive modes, each also considering both the association schemes of 2B and inert for CO_2_.

Based on the results of Table 2 for CO_2_ + DES, the predictive PC-SAFT EoS had a total *AARD%* of 63.77 and 84.65% for the inert-2B and 2B–2B modes, respectively. Therefore, on the average, the inert association scheme for CO_2_ results in lower AARD% than the 2B association scheme, most particularly, for DESs with choline chloride or tetrabutylphosphonium bromide as their HBA. However, for the majority of DESs whose, pure PC-SAFT parameters were optimized based on the density data generated by Haghbakhsh et al.‘s density model (Haghbakhsh et al., 2019), the results of the 2B association scheme had slightly lower *AARD%* values. But all in all, the results are not acceptable in these predictive modes of calculations. By considering adjusted binary interaction parameters, both of the association schemes improve significantly and produce acceptable errors. The inert-2B and 2B–2B cases resulted in total *AARD%* of 8.08% and 8.12%, respectively.

**FIGURE 4:**
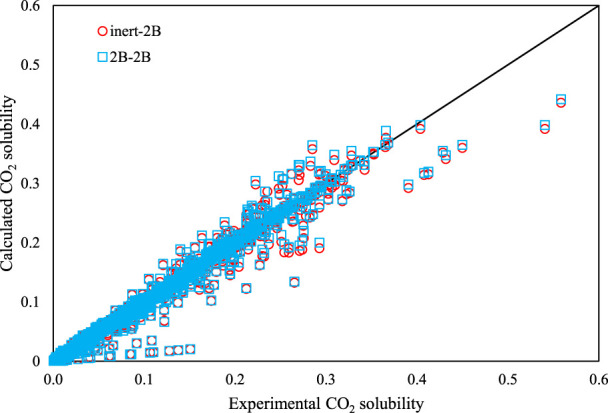
The calculated CO_2_ solubility by PC-SAFT vs. experimental values, for both association schemes of inert-2B and 2B–2B for all of the investigated DESs.

The trends between the calculated CO_2_ solubility values by the PC-SAFT versus the corresponding experimental values are presented in Figure 4 for all of the investigated DESs, using both the association schemes of inert-2B and 2B–2B. A normal behavior is observed for the PC-SAFT by noticing that most of the results are located very close to the diagonal line. Also, this figure shows that the accuracy of PC-SAFT generally decreases in the region of high CO_2_ absorption.

**FIGURE 5:**
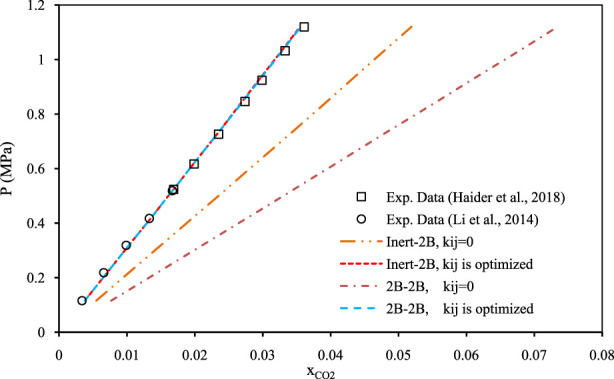
Comparison of two association schemes of PC-SAFT (inert-2B and 2B–2B) for solubility of CO_2_ in choline chloride + diethylene glycol (1:4) (Li et al., 2014; Haider et al., 2018) at the temperature of 303.15 K. The pure component PC-SAFT parameters of the DES were optimized based on experimental density data (Li et al., 2014; Haider et al., 2018).

**FIGURE 6:**
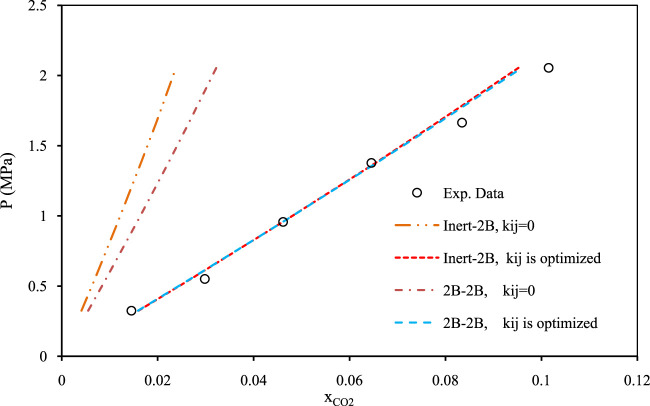
Comparison of two association schemes of PC-SAFT (inert-2B and 2B–2B) for solubility of CO_2_ in benzyltriethylammonium chloride + acetic acid (1:2) (Sarmad et al., 2017) at the temperature of 298.15 K. The pure compoent PC-SAFT parameters of the DES were optimized using the density model of Haghbakhsh et al. (2019).

**FIGURE 7:**
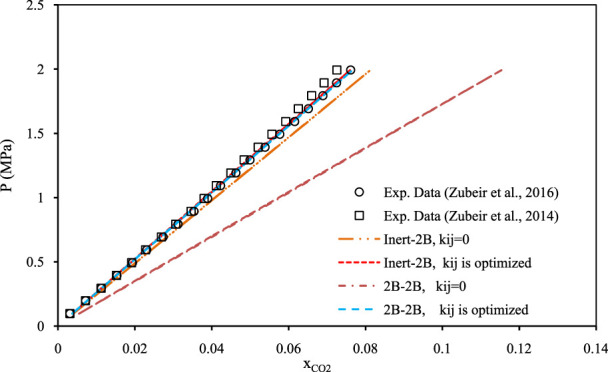
Comparison of two associations scheme of PC-SAFT (inert-2B and 2B–2B) for solubility of CO_2_ in tetraethylammonium chloride + L-lactic acid (1:2) (Zubeir et al., 2016) and (Zubeir et al., 2014) at the temperature of 308 K. The pure PC-SAFT parameters of DES were taken from the literature (Zubeir et al., 2016).

Figures 5–7 show pressure vs. CO_2_ solubility for three random DESs at various temperatures. These three DESs are considered as representatives, applying the different methods of obtaining the pure PC-SAFT parameters. In Figure 5, the pure PC-SAFT parameters of choline chloride + diethylene glycol (1:4) were optimized based on its experimental density data, while in Figure 6, the pure PC-SAFT parameters of benzyltriethylammonium chloride (BTEAC) + acetic acid (1:2) were optimized based on the general density model of Haghbakhsh et al. (Haghbakhsh et al., 2019). Figure 7 represents the phase behavior of CO_2_ with tetraethylammonium chloride (TEAC) + L-lactic acid (1:2), whose PC-SAFT parameters were taken from the literature. Figures 5–7 compare the trends of PC-SAFT-calculated phase behavior using both association schemes of inert and 2B to represent CO_2_. In all three figures, both the correlative and the predictive PC-SAFT results showed CO_2_ solubility to have a linear function of pressure, consistent with the linear trend of the experimental data. This shows that PC-SAFT, even in its predictive mode, could successfully predict the solubility trends in the presented systems, however, it does need adjustment by including binary interaction parameters in order to produce more reliable results with respect to the experimental values. The predictive PC-SAFT in Figure 6 shows overestimations of CO_2_ solubility at fixed pressures, while it shows underestimations in Figures 5, 7. This shows a normal behavior for the predictive PC-SAFT, i.e., there is no systematic underestimation or overestimation by the model, as both cases are observed. Furthermore, these three figures show that the correlative PC-SAFT presents reliable agreement with the experimental values and trends for both the carbon dioxide association schemes of 2B and inert.

For further details, the solubility of CO_2_ in different HBAs (choline chloride, allyltriphenyl phosphonium bromide, and tetrabutyl ammonium bromide) with different HBDs and various molar ratios are presented in the Supplementary section (Supplementary Figures S2–S4). It can be seen from these Supplementary figures that upon increasing the molar ratios of the glycols (ethylene glycol, diethylene glycol or triethylene glycol) as the HBD components of the investigated DESs, the CO_2_ solubility decreases. The developed PC-SAFT models, in all cases, could follow these trends quite well.

**FIGURE 8:**
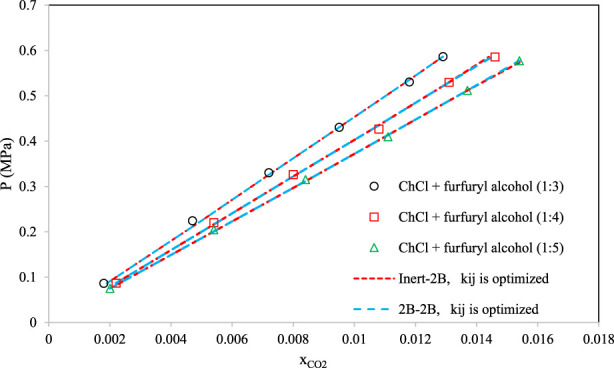
Comparison of the phase behaviors of CO_2_ with choline chloride + n-furfuryl alcohol (Lu et al., 2015) (n = 3, 4, and 5) by the correlative mode of PC-SAFT, at the temperature of 333.15 K.

**FIGURE 9:**
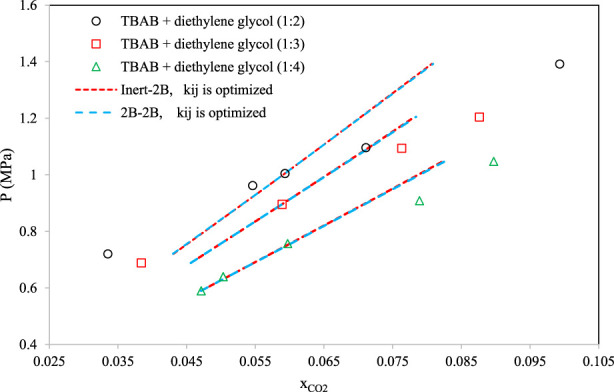
Comparison of the phase behavior of CO_2_ with 1-tetrabutyl ammonium bromide (TBAB) + n-diethylene glycol (Haider et al., 2018) (n = 2, 3, and 4) by the correlative mode of PC-SAFT, at the temperature of 303.15 K.

**FIGURE 10:**
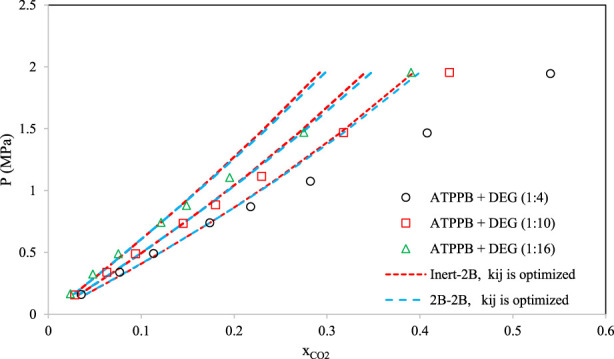
Comparison of the phase behavior of CO_2_ with 1-allyltriphenyl phosphonium bromide (ATPPB) + n-diethylene glycol (DEG) (Ghaedi et al., 2017) (n = 4, 10, and 16) by the correlative mode of PC-SAFT, at the temperature of 303.15 K.

Figures 8–10 aim to compare members of the same family of DESs (identical HBA and HBD but various molar ratios). By increasing the molar ratio (HBA with respect to HBD) in each family, the DESs show different capacities and trends for CO_2_ absorption. Figure 8 presents the correlative mode of the PC-SAFT model for choline chloride + n-furfuryl alcohol (n = 3, 4, and 5) at various molar ratios at a temperature of 333.15 K. As can be seen, the phase behavior of CO_2_ with the chosen family of DES can be calculated precisely by both of the investigated schemes. In Figure 9, the phase behavior representations of CO_2_ with 1-tetrabutyl ammonium bromide (TBAB) + n-diethylene glycol (n = 2, 3, and 4) are given at the temperature of 303.15 K. In this figure, by increasing the molar ratio of the HBD (diethylene glycol), CO_2_ absorption increases. The experimental trends are very well followed by both of the investigated association schemes. Figure 10 exhibits the CO_2_ solubility in 1-allyltriphenyl phosphonium bromide + n-diethylene glycol family (n = 4, 10, and 16) at the temperature of 303.15 K. Opposite to the DES family of Figure 9, in Figure 10, by increasing the molar ratio of diethylene glycol in this family, the solubility of CO_2_ is decreasing, which is also followed by the correlative mode of PC-SAFT with good agreement.

Based on the achieved results, the inert scheme for CO_2_, in general, shows better results compared to the 2B scheme when the predictive mode is considered (when neglecting binary interaction parameters). Despite this, the predictive mode does not give acceptable results with either of the schemes. However, when considering binary interaction parameters, both association schemes of inert and 2B for CO_2_ show trustworthy estimations with respect to the experimental trends.

## Conclusion

In contrast to previous studies focusing on a very limited number of DESs, in this study, the PC-SAFT EoS has been chosen as an associating EoS to estimate carbon dioxide solubilities in 109 deep eutectic solvents having different chemical natures over wide ranges of temperatures and pressures. This is indeed a thermodynamic challenge, considering that the DESs consist of various types of HBDs and HBAs, and at different molar ratios, resulting in complex interactions. High pressures further add to the challenge of thermodynamic modelling. Therefore, for the first time, this study gives an overview of the capabilities of this sophisticated model, as a tool for the general modelling of DES + CO_2_ phase behavior. A large and most-recent data bank, consisting of 2,542 solubility data points, is used. To obtain the pure component parameters of PC-SAFT, which are not reported in the literature, a data bank of experimental densities was also collected, consisting of a total of 62 various DESs, with 656 density data points.

The pseudo-component approach was used in this study. The association scheme of 2B was considered for the DES pseudo-components, while the association schemes of inert and 2B were both investigated for carbon dioxide. For a more extensive investigation on the capability of the PC-SAFT EoS, predictive and correlative modes were both studied. In the predictive mode, the CO_2_ solubility was calculated without considering any binary interaction parameters (*k*_*ij*_), while in the correlative mode, a binary interaction parameter was considered as a function of temperature.

One of the greatest challenges in using associating equations of state, and thus limiting their use by researchers, is the determination of the pure component parameters. This is a cause of regret, because DESs are truly associating compounds, and only models that do consider these associations are theoretically sound models for such complex systems. To assist in the more widespread use of the PC-SAFT by researchers, a simple generalized correlation is proposed in this study to estimate the PC-SAFT pure component parameters of DESs. This generalized correlation is only a function of molecular weight, and so, easily applicable to any DES. In this way, the challenging step of parameter optimization by users is eliminated. The PC-SAFT, in the predictive mode, showed total *AARD%* of 63.77 and 84.65% for the inert-2B and 2B–2B modes, respectively. But in the correlative calculations, the inert-2B and 2B–2B modes, led to total *AARD%* of 8.08% and 8.12%, respectively. The calculated solubilities by the predictive mode showed that the inert scheme for CO_2_ leads to less errors than the scheme of 2B, however, both schemes are inaccurate. By considering adjusted binary interaction parameters, the results improve significantly, with both the inert-2B and 2B–2B calculations showing reliable results with respect to the experimental trends. In its current state, DESs can still be considered as novel solvents with much unknowns. These limits also have their impact on the thermodynamic modelling of systems involving DESs. For accurate modelling, the number and strength of association bonds between carbon dioxide and the HBA or HBD molecules must be known. For example, performing NMR tests on these systems can provide valuable information. This is because systems of carbon dioxide with DESs are very complex, and in order to succeed in very accurate modeling, all the established associations in the mixture should be involved in the thermodynamic model. However, in this way, the model will become more complicated and time-consuming, but this is the cost of greater accuracy.

## Data Availability

The original contributions presented in the study are included in the article/Supplementary Material, further inquiries can be directed to the corresponding author.
