# Supplementary material for: Investigation of carbon dioxide solubility in various families of deep eutectic solvents by the PC-SAFT EoS
Source: Front Chem. 2022 Aug 9;10:909485. doi: 10.3389/fchem.2022.909485 (PMC9395712; doi:10.3389/fchem.2022.909485)
Supplement: Supplementary file 1 [file DataSheet1.pdf]

Supplementary material

Investigation of carbon dioxide solubility in various families of deep eutectic solvents by the PC-SAFT EoS

Khalil Parvaneh[1], Reza Haghbakhsh[2,3], Ana Rita C. Duarte[3], Sona Raeissi[4,*]

[1] Department of Chemical Engineering, University of Gonabad, Gonabad, Iran

[2] Department of Chemical Engineering, Faculty of Engineering, University of Isfahan, 81746-73441, Isfahan, Iran

[3] LAQV, REQUIMTE, Departamento de Química da Faculdade de Ciências e Tecnologia, Universidade Nova de Lisboa, 2829-516 Caparica, Portugal

[4] School of Chemical and Petroleum Engineering, Shiraz University, Mollasadra Ave., Shiraz 71348-51154, Iran

*** Correspondence:**

Corresponding author at: School of Chemical and Petroleum Engineering, Shiraz University, Mollasadra Ave., Shiraz 71348-51154, Iran.
Tel.: +98 71 36133704; Fax: +98 71 36474619
E-mail address: raeissi@shirazu.ac.ir

Table S1. Pressure, temperature, carbon dioxide solubility range, and number of investigated data for the studied DESs.

Abbr.	DES			Pressure range (MPa)	Temperature range (K)	Solubility range	ndp	Ref
	HBA	HBD	HBA:HBD					
DES1	acetylcholine chloride	1,2,4-triazole	1:1	0.0563-0.5932	303.15-333.15	0.0023-0.1508	28	[28]
DES2	acetylcholine chloride	guaiacol	1:3	0.0537-0.5353	293.15-323.15	0.0018-0.0256	24	[29]
DES3	acetylcholine chloride	guaiacol	1:4	0.053-0.5586	293.15-323.15	0.0017-0.0252	24	[29]
DES4	acetylcholine chloride	guaiacol	1:5	0.0486-0.5358	293.15-323.15	0.0016-0.0255	24	[29]
DES5	acetylcholine chloride	imidazole	2:3	0.0298-0.594	303.15-333.15	0.0008-0.0252	29	[28]
DES6	acetylcholine chloride	imidazole	1:2	0.0266-0.5899	303.15-333.15	0.0009-0.0269	35	[28]
DES7	acetylcholine chloride	imidazole	1:3	0.0263-0.5916	303.15-333.15	0.0008-0.0276	34	[28]
DES8	acetylcholine chloride	levulinic acid	1:3	0.0657-0.5764	303.15-333.15	0.0034-0.0383	28	[30]
DES9	alanine	lactic acid	1:1	0.00564-4.99527	298.15-328.15	0.0020-0.2780	80	[31]
DES10	alanine	malic acid	1:1	0.00563-4.994	298.15-328.15	0.0076-0.3161	81	[31]
DES11	allyltriphenyl phosphonium bromide	diethylene glycol	1:4	0.16-1.946	303.15	0.0354-0.5407	8	[32]
DES12	allyltriphenyl phosphonium bromide	diethylene glycol	1:10	0.156-1.954	303.15	0.0284-0.4318	8	[32]
DES13	allyltriphenyl phosphonium bromide	diethylene glycol	1:16	0.166-1.954	303.15	0.0237-0.3906	8	[32]
DES14	allyltriphenylphosphonium bromide	phenol	1:4	0.1827-1.3451	313.15-333.15	0.0203-0.2134	18	[33]
DES15	allyltriphenylphosphonium bromide	phenol	1:6	0.1599-1.3873	313.15-333.15	0.0245-0.1475	18	[33]
DES16	allyltriphenyl phosphonium bromide	triethylene glycol	1:4	0.142-1.95	303.15	0.0362-0.5583	8	[32]
DES17	allyltriphenyl phosphonium bromide	triethylene glycol	1:10	0.149-1.953	303.15	0.0296-0.4499	8	[32]
DES18	allyltriphenyl phosphonium bromide	triethylene glycol	1:16	0.146-1.957	303.15	0.023-0.4127	8	[32]
DES19	N-benzyl-2-hydroxy-n.n-dimethyl ethanaminium chloride	acetic acid	1:2	0.21-2.026	298.15	0.0071-0.0862	7	[34]
DES20	N-benzyl-2-hydroxy-n.n-dimethyl ethanaminium chloride	lactic acid	1:2	0.283-2.086	298.15	0.0021-0.0617	7	[34]
DES21	benzyltriethylammonium chloride	acetic acid	1:2	0.325-2.054	298.15	0.0145-0.1015	6	[34]
DES22	benzyltrimethylammonium chloride	acetic acid	1:2	0.219-2.037	298.15	0.0079-0.1291	7	[34]
DES23	benzyltrimethylammonium chloride	glycerol	1:2	0.394-2.026	298.15	0.037-0.259	6	[34]
DES24	betaine	lactic acid	1:1	0.00497-4.994	298.15-328.15	0.0022-0.3063	82	[31]
DES25	betaine	malic acid	1:1	0.00498-4.99617	318.15-328.15	0.0026-0.3048	41	[31]
DES26	choline chloride	1,2−propanediol	1:3	0.1085-0.5247	293.15-323.15	0.0019-0.0165	20	[35]
DES27	choline chloride	1,2−propanediol	1:4	0.1044-0.5256	293.15-323.15	0.0020-0.0165	20	[35]
DES28	choline chloride	1,4−butanediol	1:3	0.1109-0.5259	293.15-323.15	0.0025-0.0164	20	[35]
DES29	choline chloride	1,4−butanediol	1:4	0.1058-0.5190	293.15-323.15	0.0022-0.0154	20	[35]
DES30	choline chloride	2,3−butanediol	1:3	0.1140-0.5288	293.15-323.15	0.0026-0.0152	20	[35]
DES31	choline chloride	2,3−butanediol	1:4	0.1071-0.5125	293.15-323.15	0.0030-0.0188	20	[35]
DES32	choline chloride	citric acid	1:1	0.01-4.99	318.15-328.15	0.0036-0.3684	42	[13]
DES33	choline chloride	diethanol amine	1:6	0.5234-0.971	303.15	0.0322-0.0901	5	[36]
DES34	choline chloride	diethylene glycol	1:3	0.1128-1.112	293.15-323.15	0.0025-0.0352	27	[36,37]
DES35	choline chloride	diethylene glycol	1:4	0.1104-1.1197	293.15-323.15	0.0023-0.0361	27	[36,37]

DES36	choline chloride	ethylene glycol	1:2	0.036-6.323	303.15-343.15	0.000742-0.21561	63	[27,36,38]
DES37	choline chloride	fructose	1:1	0.01-5	308.15-318.15	0.0022-0.4038	41	[13]
DES38	choline chloride	furfuryl alcohol	1:3	0.0808-0.5864	303.15-333.15	0.0018-0.0197	24	[39]
DES39	choline chloride	furfuryl alcohol	1:4	0.0652-0.5854	303.15-333.15	0.0021-0.0228	24	[39]
DES40	choline chloride	furfuryl alcohol	1:5	0.0709-0.5774	303.15-333.15	0.002-0.0234	24	[39]
DES41	choline chloride	glycerol	1:2	0.1870-6.3470	303.15-343.15	0.0058-0.2850	40	[40]
DES42	choline chloride	guaiacol	1:3	0.0515-0.5365	293.15-323.15	0.0014-0.0215	24	[29]
DES43	choline chloride	guaiacol	1:4	0.0521-0.5459	293.15-323.15	0.0014-0.0221	24	[29]
DES44	choline chloride	guaiacol	1:5	0.0469-0.5389	293.15-323.15	0.0024-0.0148	24	[29]
DES45	choline chloride	lactic acid	1:1	0.01-4.99	298.15-328.15	0.003-0.3419	82	[13]
DES46	choline chloride	lactic acid	1:2	0.8290-9.3790	303.26-348.23	0.0248-0.0995	40	[41]
DES47	choline chloride	levulinic acid	1:3	0.0698-0.5830	303.15-333.15	0.0025-0.0302	24	[39]
DES48	choline chloride	levulinic acid	1:4	0.0600-0.5874	303.15-333.15	0.0024-0.0316	24	[39]
DES49	choline chloride	levulinic acid	1:5	0.0715-0.5810	303.15-333.15	0.0027-0.0333	24	[39]
DES50	choline chloride	malic acid	1:1	0.01-4.99	298.15-328.15	0.00204-0.3659	79	[13]
DES51	choline chloride	methyldiethanol amine	1:6	0.4456-1.096	303.15	0.1063-0.1558	6	[36]
DES52	choline chloride	methyldiethanol amine	1:7	0.5945-1.0345	303.15	0.1189-0.1986	6	[36]
DES53	choline chloride	phenol	1:2	0.099-13.3	293.15-333.15	0.0024-0.2754	44	[37,42]
DES54	choline chloride	phenol	1:3	0.1044-11.82	293.15-323.15	0.0029-0.2738	27	[37,42]
DES55	choline chloride	phenol	1:4	0.1082-12.17	293.15-323.15	0.0027-0.2927	27	[37,42]
DES56	choline chloride	triethylene glycol	1:3	0.1093-0.516	293.15-323.15	0.0052-0.0163	20	[37]
DES57	choline chloride	triethylene glycol	1:4	0.1093-0.5203	293.15-323.15	0.0033-0.028	20	[37]
DES58	choline chloride	urea	1:1.5	0.85-12.52	313.15-333.15	0.033-0.201	22	[43]
DES59	choline chloride	urea	1:2	0.0105-12.73	303.15-353.2	0.00038-0.309	78	[43,44,45]
DES60	choline chloride	urea	1:2.5	1.06-12.55	313.15-333.15	0.032-0.203	22	[43]
DES61	diethylamine hydrochloride	guaiacol	1:3	0.0451-0.5254	293.15-323.15	0.0019-0.0249	24	[29]
DES62	diethylamine hydrochloride	guaiacol	1:4	0.0496-0.5292	293.15-323.15	0.0019-0.0259	24	[29]
DES63	diethylamine hydrochloride	guaiacol	1:5	0.0488-0.5507	293.15-323.15	0.002-0.0275	24	[29]
DES64	methyltrioctylammonium chloride	decanoic acid	1:2	0.09-1.99	298.15-308.15	0.0112-0.2523	18	[46]
DES65	methyltrioctylammonium bromide	decanoic acid	1:2	0.09-1.99	298.15-323.15	0.0107-0.2568	27	[46]
DES66	methyltriphenyl phosphonium bromide	1.2-propanediol	1:4	0.22-2.026	298.15	0.0029-0.0677	7	[34]
DES67	methyltriphenyl phosphonium bromide	acetic acid	1:4	0.173-2.014	298.15	0.0086-0.2653	8	[34]
DES68	methyltriphenyl phosphonium bromide	ethylene glycol	1:3	0.192-2.01	298.15	0.0061-0.0456	6	[34]
DES69	methyltriphenyl phosphonium bromide	glycerol	1:4	0.161-2.026	298.15	0.00130-0.04025	6	[34]
DES70	methyltriphenyl phosphonium bromide	levulinic acid	1:3	0.301-2.068	298.15	0.0042-0.1082	7	[34]
DES71	tetrabutylammonium chloride	acetic acid	1:2	0.348-2.002	298.15	0.0238-0.1577	6	[34]
DES72	tetrabutylammonium chloride	decanoic acid	1:2	0.09-1.99	298.15-323.15	0.0086-0.2394	36	[46]
DES73	tetrabutylammonium chloride	l-lactic acid	1:2	0.0930-1.9920	298.00-318.00	0.0056-0.1551	76	[25,26]
DES74	tetrabutylammonium chloride	l-lactic acid	1:3	0.0940-1.9930	298.00-318.00	0.0054-0.1403	40	[25]
DES75	tetrabutylammonium chloride	levulinic acid	1:3	0.0632-0.5916	303.15-333.15	0.0040-0.0453	28	[30]
DES76	tetrabutylammonium bromide	acetic acid	1:2	0.388-2.011	298.15	0.0198-0.1429	5	[34]
DES77	tetrabutyl ammonium bromide	ethylene glycol	1:2	0.412-1.2791	303.15	0.0151-0.0537	5	[36]
DES78	tetrabutyl ammonium bromide	ethylene glycol	1:3	0.5025-1.2491	303.15	0.0167-0.0519	6	[36]
DES79	tetrabutyl ammonium bromide	ethylene glycol	1:4	0.5428-1.3742	303.15	0.0188-0.0496	6	[36]
DES80	tetrabutyl ammonium bromide	diethanol amine	1:6	0.6105-1.0112	303.15	0.0464-0.1019	5	[36]
DES81	tetrabutyl ammonium bromide	diethylene glycol	1:2	0.7208-1.3925	303.15	0.0335-0.0993	5	[36]

DES82	tetrabutyl ammonium bromide	diethylene glycol	1:3	0.6885-1.205	303.15	0.0384-0.0876	4	[36]
DES83	tetrabutyl ammonium bromide	diethylene glycol	1:4	0.59-1.0477	303.15	0.0471-0.0897	5	[32]
DES84	tetrabutylammonium bromide	ethanolamine	1:6	0.351-2.021	298.15	0.04141-0.21475	6	[34]
DES85	tetrabutylammonium bromide	ethanolamine	1:7	0.381-2.04	298.15	0.04759-0.22001	6	[34]
DES86	tetrabutylammonium bromide	levulinic acid	1:3	0.0702-0.5864	303.15-333.15	0.0038-0.0432	26	[30]
DES87	tetrabutylphosphonium bromide	phenol	1:4	0.1638-1.5785	313.15-333.15	0.0184-0.2048	18	[33]
DES88	tetrabutylphosphonium bromide	diethylene glycol	1:4	0.0944-1.3981	313.15-333.15	0.013-0.2123	18	[33]
DES89	tetraethylammonium chloride	acetic acid	1:2	0.281-2.018	298.15	0.0135-0.1008	6	[34]
DES90	tetraethylammonium chloride	acetic acid	1:3	0.397-2.016	298.15	0.0108-0.0961	6	[34]
DES91	tetraethylammonium chloride	l-lactic acid	1:2	0.0940-1.9930	298.00-318.00	0.0031-0.0761	87	[25,26]
DES92	tetraethylammonium chloride	levulinic acid	1:3	0.0661-0.5854	303.15-333.15	0.0028-0.0340	28	[30]
DES93	tetraethylammonium chloride	octanoic acid	1:3	0.353-2.018	298.15	0.0229-0.1721	6	[34]
DES94	tetraethylammonium bromide	levulinic acid	1:3	0.0687-0.5878	303.15-333.15	0.0030-0.0324	28	[30]
DES95	tetramethylammonium chloride	acetic acid	1:4	0.294-2.096	298.15	0.0083-0.0984	6	[34]
DES96	tetramethylammonium chloride	l-lactic acid	1:2	0.0930-1.9930	298.00-318.00	0.0023-0.0618	85	[25,26]
DES97	tetraoctylammonium bromide	decanoic acid	1:2	0.09-1.99	298.15-323.15	0.0114-0.2835	27	[46]
DES98	tetraoctylammonium chloride	decanoic acid	1:1.5	0.09-1.99	298.15-323.15	0.0122-0.2995	33	[46]
DES99	tetraoctylammonium chloride	decanoic acid	1:2	0.09-1.99	298.15-323.15	0.0117-0.2843	36	[46]
DES100	tetrapropylammonium chloride	acetic acid	1:6	0.35-2.03	298.15	0.0204-0.1252	6	[34]
DES101	tetrapropylammonium chloride	ethanolamine	1:4	0.481-2.009	298.15	0.0305-0.1174	6	[34]
DES102	tetrapropylammonium chloride	ethanolamine	1:7	0.357-2.019	298.15	0.1221-0.2225	6	[34]
DES103	triethylmethylammonium chloride	acetic acid	1:2	0.198-1.837	298.15	0.0073-0.0963	7	[34]
DES104	triethylmethylammonium chloride	ethylene glycol	1:2	0.138-1.345	298.15	0.0057-0.0544	6	[34]
DES105	triethylmethylammonium chloride	glycerol	1:2	0.15-1.648	298.15	0.0019-0.0462	5	[34]
DES106	triethylmethylammonium chloride	lactic acid	1:2	0.143-1.863	298.15	0.0052-0.0556	6	[34]
DES107	triethylmethylammonium chloride	levulinic acid	1:2	0.136-1.617	298.15	0.0072-0.0727	5	[34]
DES108	N, N, N-trimethyl-methanaminium chloride	phenol	1:3	2.36-12.17	313.15	0.0945-0.2623	7	[42]
DES109	N, N, N-triethyl-ethanaminium chloride	phenol	1:3	2.12-11.17	313.15	0.0746-0.233	7	[42]
						**Total data no.**	**2542**	

ndp is the number of data
Abbr. is the abbreviation

Table S2. Temperature range, experimental density range, number of investigated data, and the corresponding references.

Abbr.	Temperature range (K)	Exp. density range (kg/m^3)	ndp	Ref.
DES1	303.15-363.15	1100.2-1129.3	7	[1]
DES2	293.15-323.15	1133.8-1149.6	4	[2]
DES3	293.15-323.15	1131.7-1149.3	4	[2]
DES4	293.15-323.15	1135.1-1152.7	4	[2]
DES5	303.15-363.15	1095.5-1125.6	7	[1]
DES6	303.15-363.15	1089.5-1120.6	7	[1]
DES7	303.15-363.15	1128.5-1155.7	7	[1]
DES8	303.15-343.15	1106.85-1144.4	15	[3,4]
DES9	293.15-363.15	1239-1287	15	[5]
DES10	298.15-363.15	1365-1415	14	[5]
DES11	293.15-343.15	1164.2-1201.1	11	[6]
DES12	293.15-343.15	1123.3-1159.9	11	[6]
DES13	293.15-343.15	1109.9-1146.3	11	[6]
DES14	293.15-333.15	1144.3-1173	5	[7]
DES15	293.15-333.15	1129.1-1158.2	5	[7]
DES16	293.15-343.15	1153.9-1190.8	11	[6]
DES17	293.15-343.15	1121.7-1159.2	11	[6]
DES18	293.15-343.15	1108.4-1146.2	11	[6]
DES24	293.15-363.15	1167-1211	15	[5]
DES25	318.15-363.15	1271-1301	10	[5]
DES26	293.15-323.15	1046.7-1066.6	4	[8]
DES27	293.15-323.15	1048-1066.3	4	[8]
DES28	293.15-343.15	1025.3-1052.4	13	[8,9]
DES29	293.15-343.15	1018.3-1044.5	13	[8,9]
DES30	293.15-343.15	1012.8-1046.8	13	[8,9]
DES31	293.15-343.15	1003.3-1035	13	[8,9]
DES34	293.15-323.15	1108.3-1122.4	4	[10]
DES35	293.15-323.15	1103.2-1120.3	4	[10]
DES36	278.15-368.15	1074.4-1127.5	72	[11-16]
DES38	303.15-333.15	1112-1131.8	4	[17]
DES39	303.15-333.15	1109.9-1131.5	4	[17]
DES40	303.15-333.15	1109.5-1130.9	4	[17]
DES41	283.15-368.15	1149.5-1200.1	32	[11,12,18]
DES42	293.15-323.15	1134.1-1153.3	4	[2]
DES43	293.15-323.15	1132.4-1155.6	4	[2]
DES44	293.15-323.15	1128-1149.7	4	[2]
DES46	283.15-363.15	1137-1179.3	21	[19,20]
DES47	293.15-343.15	1106.48-1142	15	[17,4]
DES48	303.15-333.15	1113.1-1134.1	4	[17]
DES49	303.15-333.15	1113-1133.7	4	[17]
DES53	293.15-333.15	1065.3-1099.5	22	[10,21-23]
DES54	293.15-323.15	1077.13-1094.8	13	[10,21,23]
DES55	293.15-323.15	1072.92-1091.8	13	[10,21,23]
DES56	293.15-323.15	1112.4-1133.1	4	[10]
DES57	293.15-323.15	1103.2-1120.3	4	[10]
DES59	293.15-363.15	1155.4-1200.1	33	[24-27]
DES61	293.15-323.15	1075.8-1095.4	4	[2]

DES62	293.15-323.15	1084.1-1102.4	4	[2]
DES63	293.15-323.15	1083.5-1106.1	4	[2]
DES64	288.15-323.15	880.7-902.7	8	[28]
DES65	288.15-323.15	925.8-948.9	8	[28]
DES68	298.15-368.15	1201.3-1250.1	15	[11]
DES69	298.15-368.15	1238.5-1288.9	15	[11]
DES72	288.15-323.15	901-923.2	8	[28]
DES75	293.15-343.15	998.91-1034.53	15	[3,4]
DES86	293.15-343.15	1064.68-1100.89	15	[3,4]
DES87	293.15-333.15	1045.7-1073.4	5	[7]
DES88	293.15-333.15	1062.5-1089.1	5	[7]
DES92	293.15-343.15	1062.29-1097.41	15	[3,4]
DES94	293.15-343.15	1139.74-1177.35	15	[3,4]
DES108	303.15-323.15	1059.64-1064.12	3	[23]
DES109	303.15-323.15	1037.66-1050.21	3	[23]
		**Total data no.**	**656**	

Table S3. The temperature range, liquid density range, and number of density data points of those DESs of this study whose densities were generated by the general density model of Haghbakhsh et al. [6].

Abbr.	Temperature range (K)	Density range (Kg/m^3)	ndp	Ref
DES19	283.15-363.15	1094.4-1131.5	17	Density model [6]
DES20	283.15-363.15	1166.3-1203.4	17	Density model [6]
DES21	283.15-363.15	1057.4-1094.5	17	Density model [6]
DES22	283.15-363.15	1053.8-1090.9	17	Density model [6]
DES23	283.15-363.15	1156.9-1194.0	17	Density model [6]
DES32	283.15-363.15	1230.1-1267.2	17	Density model [6]
DES33	283.15-363.15	1130.4-1167.5	17	Density model [6]
DES37	283.15-363.15	1229.3-1266.5	17	Density model [6]
DES45	283.15-363.15	1102.3-1139.4	17	Density model [6]
DES50	283.15-363.15	1179.1-1216.2	17	Density model [6]
DES51	283.15-363.15	1099.0-1136.1	17	Density model [6]
DES52	283.15-363.15	1100.8-1137.9	17	Density model [6]
DES58	283.15-363.15	1102.5-1139.6	17	Density model [6]
DES60	283.15-363.15	1120.8-1158.0	17	Density model [6]
DES66	283.15-363.15	1149.4-1186.5	17	Density model [6]
DES67	283.15-363.15	1118.7-1155.8	17	Density model [6]
DES70	283.15-363.15	1176.9-1214.0	17	Density model [6]
DES71	283.15-363.15	1027.7-1064.8	17	Density model [6]
DES76	283.15-363.15	1040.9-1078.1	17	Density model [6]
DES77	283.15-363.15	1066.3-1103.4	17	Density model [6]
DES78	283.15-363.15	1077.8-1114.9	17	Density model [6]
DES79	283.15-363.15	1084.4-1121.6	17	Density model [6]
DES80	283.15-363.15	1119.8-1157.0	17	Density model [6]
DES81	283.15-363.15	1075.7-1112.8	17	Density model [6]
DES82	283.15-363.15	1092.1-1129.2	17	Density model [6]
DES83	283.15-363.15	1101.6-1138.8	17	Density model [6]
DES84	283.15-363.15	1065.3-1102.4	17	Density model [6]
DES85	283.15-363.15	1067.1-1104.2	17	Density model [6]
DES89	283.15-363.15	1008.6-1045.7	17	Density model [6]
DES90	283.15-363.15	1023.3-1060.4	17	Density model [6]
DES93	283.15-363.15	1030.9-1068.1	17	Density model [6]
DES94	293.15-343.15	1139.74-1177.35	15	Density model [6]
DES95	283.15-363.15	1026.6-1063.7	17	Density model [6]
DES100	283.15-363.15	1046.8-1083.9	17	Density model [6]
DES101	283.15-363.15	1046.5-1083.7	17	Density model [6]
DES102	283.15-363.15	1059.0-1096.1	17	Density model [6]
DES103	283.15-363.15	1005.8-1043.0	17	Density model [6]
DES104	283.15-363.15	1036.3-1073.5	17	Density model [6]
DES105	283.15-363.15	1117.1-1154.2	17	Density model [6]
DES106	283.15-363.15	1093.1-1130.2	17	Density model [6]
DES107	283.15-363.15	1081.9-1119.0	17	Density model [6]

Table S4. The calculated critical properties and acentric factors according to the modified Lydersen-Joback-Reid approach and the Lee-Kesler mixing rules, for those DESs whose density data were generated by the general density model of Haghbakhsh et al. [6].

DES number	T_c (K)	P_c (bar)	V_c (cm^3/mol)	ω
DES19	649.19	41.23	306.97	0.6592
DES20	730.79	34.77	367.30	0.9447
DES21	631.92	38.40	330.80	0.5730
DES22	614.15	43.69	286.78	0.5309
DES23	706.55	31.47	360.53	1.1450
DES32	835.65	29.36	439.76	1.2310
DES33	693.44	29.66	384.11	1.0925
DES37	806.29	26.73	417.18	1.4604
DES45	655.93	35.43	334.27	0.8629
DES50	742.56	32.60	377.18	1.0746
DES51	671.07	28.43	396.67	1.0401
DES52	672.11	28.46	395.77	1.0459
DES58	641.30	46.02	271.37	0.6619
DES60	646.90	52.32	242.44	0.6431
DES66	707.97	35.36	354.15	0.9149
DES67	669.45	47.59	284.49	0.5560
DES70	827.47	33.30	478.26	0.6945
DES71	629.43	30.86	399.88	0.6435
DES76	642.33	31.17	403.40	0.6476
DES77	655.37	27.14	420.87	0.9518
DES78	637.85	31.09	355.99	0.9620
DES79	628.06	34.11	318.69	0.9682
DES80	717.72	25.53	458.85	1.1080
DES81	712.32	23.19	528.73	0.9816
DES82	700.82	25.45	471.34	0.9956
DES83	694.28	27.04	437.89	1.0040
DES84	613.80	39.21	295.71	0.7443
DES85	610.58	40.76	283.29	0.7417
DES89	576.34	41.18	285.23	0.5335
DES90	575.84	46.45	252.60	0.5343
DES93	684.70	25.22	525.99	0.6760
DES95	560.77	58.35	198.03	0.5017
DES100	586.42	50.32	235.34	0.5599
DES101	601.93	38.03	301.87	0.7188
DES102	596.93	43.34	262.53	0.7205
DES103	569.58	43.19	270.14	0.5193
DES104	581.89	37.37	285.44	0.8235
DES105	660.01	31.10	342.57	1.1334
DES106	648.05	36.47	328.13	0.8049
DES107	682.37	33.03	405.78	0.6381

Table S5. The values of segment (m), segment energy (ε/k) and reduced critical temperature (T^*_c) for PC-SAFT parameters of all investigated DESs in this study

Abbr.	m_i	T_c (K)	ε_i/k (K)	T^*_c
DES1	3.6803	682.86	420.74	1.623
DES2	9.4497	714.77	309.65	2.308
DES3	8.9837	716.52	327.52	2.188
DES4	7.0125	717.76	340.12	2.110
DES5	10.3775	632.21	343.99	1.838
DES6	9.3189	641.14	336.29	1.907
DES7	8.7806	635.41	339.50	1.872
DES8	9.7048	731.12	318.15	2.298
DES9	3.2933	696.67	381.91	1.824
DES10	3.4444	785.68	355.21	2.212
DES11	3.4128	756.24	335.16	2.256
DES12	3.4170	709.60	358.76	1.978
DES13	3.3869	696.23	354.27	1.965
DES14	3.5068	747.06	332.58	2.246
DES15	3.4600	722.02	332.86	2.169
DES16	3.5507	817.24	327.82	2.493
DES17	3.5021	776.85	327.72	2.370
DES18	3.4751	765.16	332.31	2.303
DES19	7.5169	649.19	359.28	1.807
DES20	6.9359	730.79	349.74	2.090
DES21	7.1671	631.92	342.91	1.843
DES22	6.6122	614.15	380.24	1.615
DES23	5.7468	706.55	407.43	1.734
DES24	3.1064	654.79	387.70	1.689
DES25	3.4218	741.58	395.86	1.873
DES26	3.3837	620.93	348.92	1.780
DES27	4.3270	621.24	350.00	1.775
DES28	4.3312	637.97	391.45	1.630
DES29	3.6904	639.21	390.63	1.636
DES30	4.6418	640.23	393.62	1.627
DES31	5.3061	641.64	332.52	1.930
DES32	6.6024	835.65	348.71	2.396
DES33	7.8434	693.44	356.71	1.944
DES34	6.3107	658.58	345.30	1.907
DES35	4.6314	661.22	361.14	1.831
DES36	3.7883	602.00	320.39	1.879
DES37	6.2709	806.29	368.99	2.185
DES38	8.4619	647.43	345.22	1.875
DES39	7.5213	649.45	347.78	1.867
DES40	6.7674	650.84	354.09	1.838
DES41	5.3496	680.67	310.00	2.196
DES42	9.1060	696.74	351.98	1.979
DES43	7.0991	702.17	352.35	1.993
DES44	5.5588	705.84	341.30	2.068
DES45	6.1204	655.93	396.20	1.656
DES46	5.1851	668.77	284.13	2.354
DES47	5.4774	712.77	323.72	2.202
DES48	3.7486	719.21	291.86	2.464

DES49	3.0017	723.53	280.52	2.579
DES50	6.3275	742.56	377.26	1.968
DES51	7.1170	671.07	347.81	1.929
DES52	7.1606	672.11	355.33	1.892
DES53	6.3374	647.03	257.25	2.515
DES54	5.3589	650.57	241.00	2.699
DES55	5.1909	652.77	229.25	2.847
DES56	9.0710	712.69	330.58	2.156
DES57	8.7878	718.89	317.32	2.265
DES58	3.4365	641.30	353.06	1.816
DES59	3.3586	644.44	360.18	1.789
DES60	3.3674	646.90	383.90	1.685
DES61	10.5724	680.55	323.10	2.106
DES62	10.5806	689.18	362.07	1.903
DES63	10.5485	694.99	361.91	1.920
DES64	11.0243	833.57	304.89	2.734
DES65	11.0359	845.08	305.57	2.766
DES66	6.3126	707.97	372.90	1.899
DES67	6.0238	669.45	358.31	1.868
DES68	6.8842	708.03	387.74	1.826
DES69	9.5777	783.62	347.43	2.255
DES70	6.7541	827.47	342.42	2.417
DES71	6.3977	629.43	378.56	1.663
DES72	7.8481	764.40	337.11	2.268
DES73*	11.7300	710.53	303.76	2.339
DES74*	10.5328	704.67	338.00	2.085
DES75	8.7261	745.52	266.40	2.799
DES76	8.9204	642.33	330.34	1.944
DES77	7.2637	655.37	366.66	1.787
DES78	6.8383	637.85	341.56	1.867
DES79	6.4777	628.06	387.90	1.619
DES80	6.1411	717.72	357.80	2.006
DES81	9.5816	712.32	321.28	2.217
DES82	8.3764	700.82	339.72	2.063
DES83	7.2833	694.28	357.95	1.940
DES84	6.4463	613.80	356.45	1.722
DES85	6.4289	610.58	382.36	1.597
DES86	8.6644	755.70	252.88	2.988
DES87	3.9823	690.87	365.60	1.890
DES88	4.0299	700.29	364.79	1.920
DES89	8.0400	576.34	329.75	1.748
DES90	7.2645	575.84	365.81	1.574
DES91*	3.2607	655.16	599.88	1.092
DES92	5.5147	703.07	280.15	2.510
DES93	8.8536	684.70	344.45	1.988
DES94	5.2004	714.95	350.74	2.038
DES95	4.9495	560.77	379.67	1.477
DES96*	2.9824	625.90	506.01	1.237
DES97**	15.4820	904.74	317.42	2.850
DES98**	14.8000	922.78	382.09	2.415
DES99**	15.3220	894.25	307.11	2.912
DES100	7.2315	586.42	359.78	1.630

DES101	6.1747	601.93	352.67	1.707
DES102	5.8932	596.93	340.80	1.752
DES103	6.5848	569.58	369.45	1.542
DES104	6.2763	581.89	364.94	1.594
DES105	6.8388	660.01	353.41	1.868
DES106	6.2883	648.05	396.64	1.634
DES107	6.7412	682.37	359.07	1.900
DES108	3.7112	618.69	404.08	1.531
DES109	8.9137	640.94	335.54	1.910

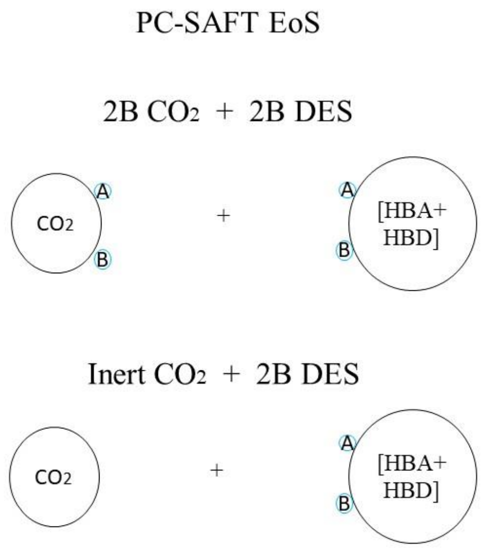

Figure S1. Schematic presentation of considered association schemes for CO_2 and DES in this study.

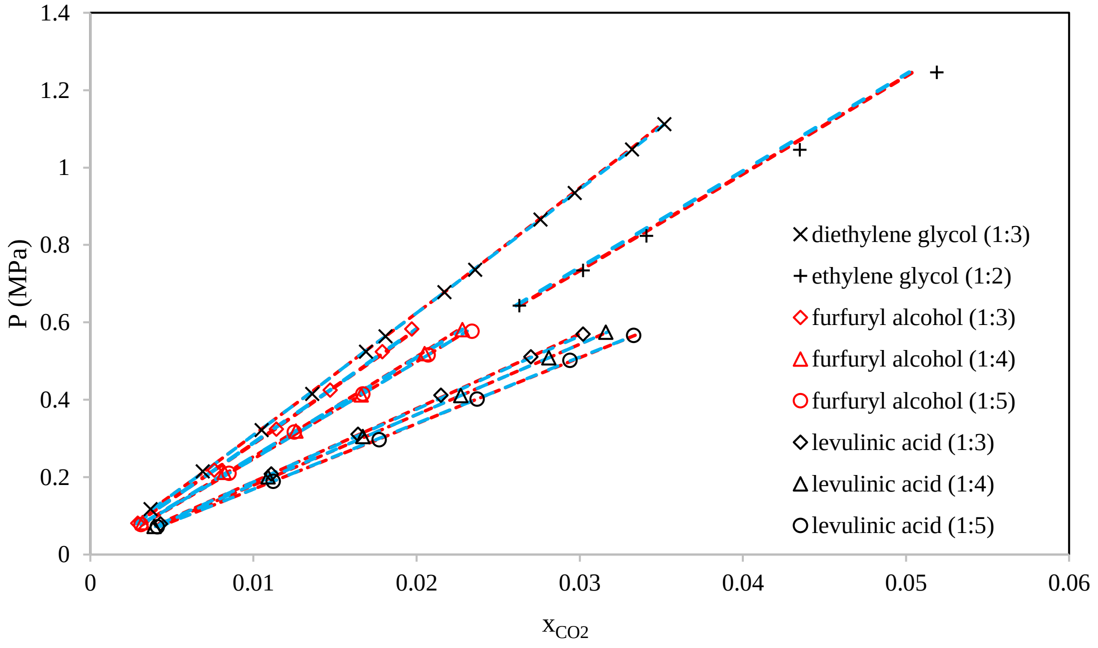

Figure S2. Comparison of two association schemes of PC-SAFT (inert-2B and 2B-2B) for the solubility of CO_2 in DESs of choline chloride with various HBDs and various molar ratios [17, 30-32] at the temperature of 303.15 K.

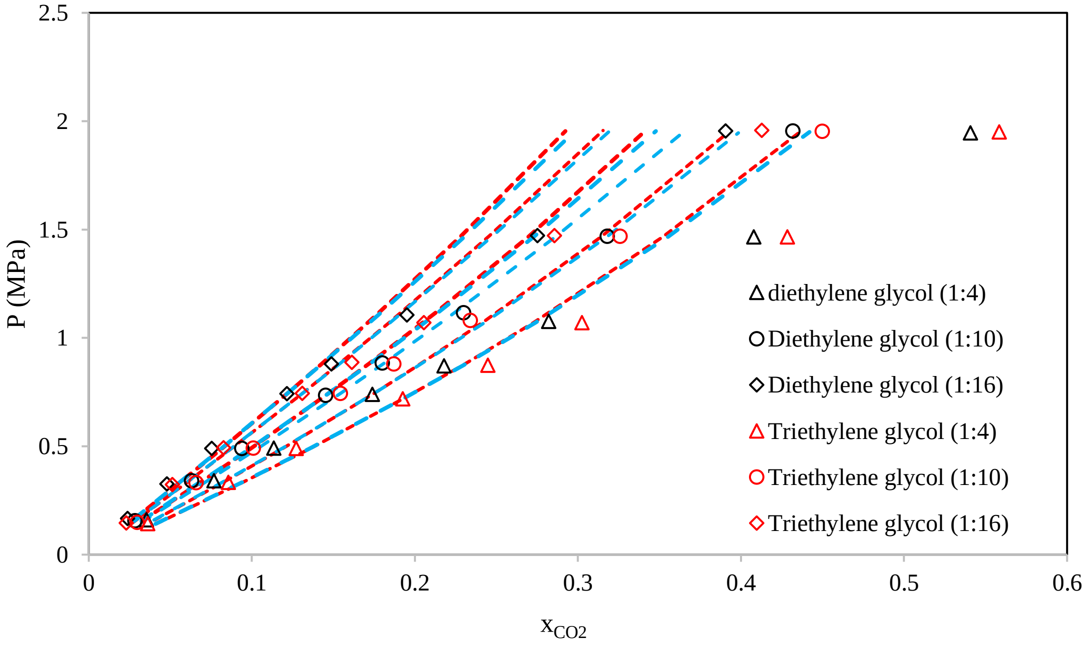

Figure S3. Comparison of two association schemes of PC-SAFT (inert-2B and 2B-2B) for the solubility of CO_2 in DESs of allyltriphenyl phosphonium bromide (ATPPB) with various HBDs and various molar ratios [33] at the temperature of 303.15 K.

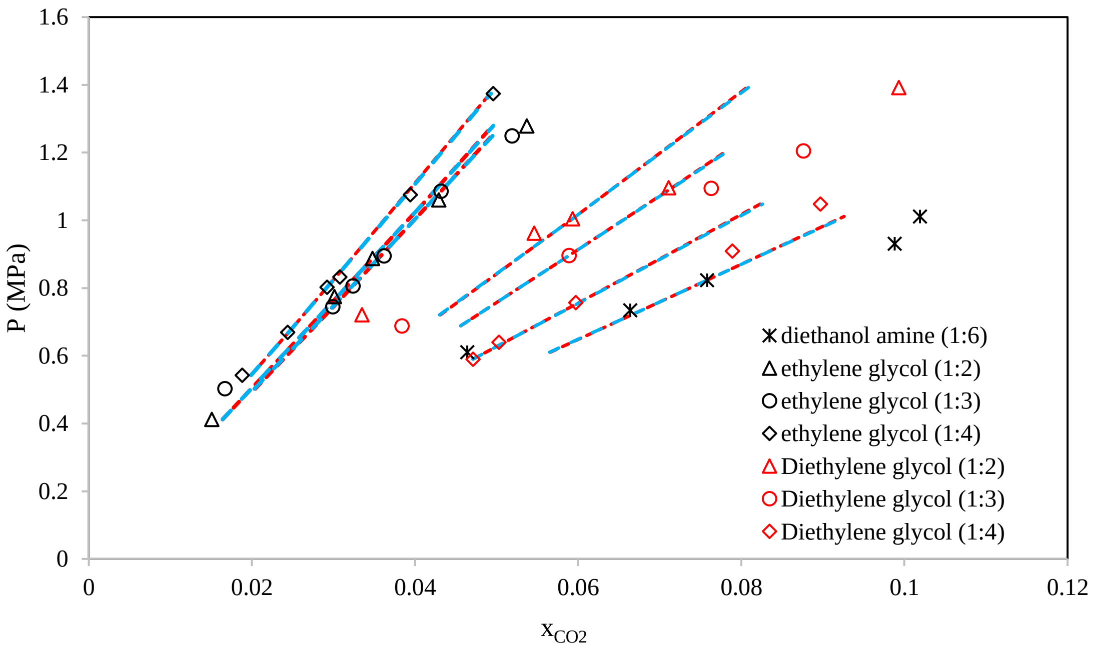

Figure S4. Comparison of two association schemes of PC-SAFT (inert-2B and 2B-2B) for the solubility of CO_2 in DESs of tetrabutyl ammonium bromide (TBAB) with various HBDs and various molar ratios [31] at the temperature of 303.15 K.

References

Reference list is provided in the main text of this article.
